# Machine-learning assisted discovery unveils novel interplay between gut microbiota and host metabolic disturbance in diabetic kidney disease

**DOI:** 10.1080/19490976.2025.2473506

**Published:** 2025-03-06

**Authors:** I-Wen Wu, Yu-Chieh Liao, Tsung-Hsien Tsai, Chieh-Hua Lin, Zhao-Qing Shen, Yun-Hsuan Chan, Chih-Wei Tu, Yi-Ju Chou, Chi-Jen Lo, Chi-Hsiao Yeh, Chun-Yu Chen, Heng-Chih Pan, Heng-Jung Hsu, Chin-Chan Lee, Mei-Ling Cheng, Wayne Huey-Herng Sheu, Chi-Chun Lai, Huey-Kang Sytwu, Ting-Fen Tsai

**Affiliations:** a Department of Nephrology, Chang Gung Memorial Hospital, Keelung, Taiwan; b Community Medicine Research Center, Chang Gung Memorial Hospital, Keelung, Taiwan; c Institute of Population Health Sciences, National Health Research Institutes, Miaoli, Taiwan; d Advanced Tech BU, Acer Inc, New Taipei City, Taiwan; e Department of Life Sciences and Institute of Genome Sciences, National Yang Ming Chiao Tung University, Taipei, Taiwan; f Institute of Molecular and Genomic Medicine, National Health Research Institutes, Miaoli, Taiwan; g Metabolomics Core Laboratory, Healthy Aging Research Center, Chang Gung University, Taoyuan, Taiwan; h College of Medicine, Chang Gung University, Taoyuan, Taiwan; i Department of Thoracic and Cardiovascular Surgery, Chang Gung Memorial Hospital, Taoyuan, Taiwan; j Clinical Metabolomics Core Laboratory, Chang Gung Memorial Hospital, Taoyuan, Taiwan; k Department of Biomedical Sciences, College of Medicine, Chang Gung University (MLC), Taoyuan, Taiwan; l Division of Endocrinology and Metabolism, Department of Internal Medicine, Taipei Veterans General Hospital, Taipei, Taiwan; m School of Medicine, National Yang Ming Chiao Tung University, Taipei, Taiwan; n Department of Ophthalmology, Chang Gung Memorial Hospital, Keelung, Taiwan; o National Institute of Infectious Diseases and Vaccinology, National Health Research Institutes, Miaoli, Taiwan; p Department & Graduate Institute of Microbiology and Immunology, National Defense Medical Center, Taipei, Taiwan; q Center for Healthy Longevity and Aging Sciences, National Yang Ming Chiao Tung University, Taipei, Taiwan

**Keywords:** Diabetic kidney disease, microbiota, machine learning, branched-chain amino acids

## Abstract

Diabetic kidney disease (DKD) is a serious healthcare dilemma. Nonetheless, the interplay between the functional capacity of gut microbiota and their host remains elusive for DKD. This study aims to elucidate the functional capability of gut microbiota to affect kidney function of DKD patients. A total of 990 subjects were enrolled consisting of a control group (*n* = 455), a type 2 diabetes mellitus group (DM, *n* = 204), a DKD group (*n* = 182) and a chronic kidney disease group (CKD, *n* = 149). Full-length sequencing of 16S rRNA genes from stool DNA was conducted. Three findings are pinpointed. Firstly, new types of microbiota biomarkers have been created using a machine-learning (ML) method, namely relative abundance of a microbe, presence or absence of a microbe, and the hierarchy ratio between two different taxonomies. Four different panels of features were selected to be analyzed: (i) DM *vs*. Control, (ii) DKD *vs*. DM, (iii) DKD *vs*. CKD, and (iv) CKD *vs*. Control. These had accuracy rates between 0.72 and 0.78 and areas under curve between 0.79 and 0.86. Secondly, 13 gut microbiota biomarkers, which are strongly correlated with anthropometric, metabolic and/or renal indexes, concomitantly identified by the ML algorithm and the differential abundance method were highly discriminatory. Finally, the predicted functional capability of a DKD-specific biomarker, *Gemmiger* spp. is enriched in carbohydrate metabolism and branched-chain amino acid (BCAA) biosynthesis. Coincidentally, the circulating levels of various BCAAs (L-valine, L-leucine and L-isoleucine) and their precursor, L-glutamate, are significantly increased in DM and DKD patients, which suggests that, when hyperglycemia is present, there has been alterations in various interconnected pathways associated with glycolysis, pyruvate fermentation and BCAA biosynthesis. Our findings demonstrate that there is a link involving the gut-kidney axis in DKD patients. Furthermore, our findings highlight specific gut bacteria that can acts as useful biomarkers; these could have mechanistic and diagnostic implications.

## Introduction

Diabetic kidney disease (DKD) is the leading cause of end-stage kidney disease worldwide.^1^ Hyperglycemia causes a cascade of molecular changes that triggers increases in oxidative stress, together with higher levels of inflammatory cytokines, various growth factors, and a number of profibrotic substances. An imbalance in these important factors results in structural and functional changes to various molecular and intracellular moieties. In turn, this leads to mesangial expansion, podocyte injury, tubulointerstitial fibrosis and glomerulosclerosis.^2^ Eventually, this metabolic disarrangement further aggravates host homeostasis, which leads to kidney damage.^3^

The gut microbiota is an extensive endogenous repertoire of microorganisms that produce many gut metabolites that have physiological functions.^4–6^ The uremic milieu, disease-related dietary modification and pharmaceutical interventions in DKD patients profoundly affect the functioning of the intestinal barrier, the species that are present in the gut microbiota, and the production of metabolites within the gut.^7–10^ Significant microbiota architectural changes, as well as functional alterations, have been extensively reported to be present in chronic kidney disease (CKD) and DKD patients.^11–13^ Currently, there are unmet challenges regarding the inter-comparability of such studies and the generalizability of their findings; this has impeded progress in our understanding of the place of the microbiome in medicine.^14^ The complexity of the high-dimensional datasets generated by full-length bacterial 16S rRNA gene sequencing and the meaningful analysis of the resulting metagenome sequences remains challenging when addressed by computational methods. Compositional tables are usually used to identify the relative abundances of specific species, but each sample contains a huge number of features, many of which are sparse in terms of numbers; furthermore, there are excessive zero counts.^15^ Typically, the application of a prevalence percentage filter, the use of log-transformations, applying a staying-in-the-simplex approach, or using ratios calculations are the normal approaches to solving the above problems.^16^ In spite of technological advances and the use of broad metadata collections in published studies, further analytical refinement, together with improved study design and increased sample size, are warranted in order to facilitate standardization of the methods used and the translation of study findings into useful clinical findings.

Machine learning (ML) has emerged as one of the artificial intelligence (AI) methodologies that can be used during microbiome research. ML provides an innovative and integrative method that can assist traditional analysis procedures in order to improve prediction. Rigorous feature selection and extraction procedures are able to help overcome the dimensional complexity. The use of different ML algorithms during the analysis of the microbiome composition can also enhance microbial biomarker classification, phenotype prediction, possible host interactions and potential endogenous component interactions.^12,15^

In this study, we adopt an integrative approach involving the use of ML together with differential abundance methods in order to investigate the composition and diversity of gut microbiota by analyzing full-length 16S rRNA gene sequencing. The fecal samples came from a relatively large cohort of diabetic patients with diverse levels of renal function, as well as from controls subjects with normal renal function. In addition, we incorporate metabolite datasets from blood that targeted lipidomic profiles in order to get insights into the possible interplay between the host metabolism and the host gut microbes. Moreover, the functional capabilities of various metabolic pathways that are potentially associated with DKD-prone microbial metabolism were explored in order to provide a foundation for translating these research findings into personalized therapeutic strategies.

## Materials and methods

### Patient setting and clinical samples

We enrolled participants who were members of the Northeastern Taiwan Community Medicine Research Cohort (ClinicalTrials.gov: NCT04839796) into this study.^17–19^ Specifically, this study recruited community inhabitants aged greater than 30 years old between August 2013 and November 2022. Subjects who were pregnant, who were undergoing dialysis therapy, and/or who had undergone renal transplantation, were excluded. All participants gave signed written informed consent. This study protocol conforms to the ethical guidelines of the 1975 Declaration of Helsinki and was approved by the Institutional Review Board of Chang Gung Medical Foundation (IRB No: 201800802B0, 202000077B0A3, 201800273B0C602, 202002535B0).

### Disease definitions

After enrollment, the participants were classified into various different disease groups according to the following clinical definitions.^17–19^ Type 2 DM was defined as a fasting glucose of ≥126 mg/dL, a glycosylated hemoglobin ≥6.5, or the use of hypoglycemic medication. CKD was defined using the National Kidney Foundation: Kidney Disease Outcomes Quality Initiative classification. These subjects had persistent proteinuria or an eGFR of less than 60 mL/min/1.73 m^2^, as determined by the abbreviated Modification of Diet in Renal Disease equation.^20^ Proteinuria was present if the protein to creatinine ratio was ≥150 mg/g or the urine albumin to creatinine ratio was ≥30 mg/g. DKD was diagnosed if subjects fulfilled both the DM criteria and the CKD criteria at the same time. Patients were not obese, did not have DM, CKD, DKD, or an acute illness were classified into the control group. Examination of a subject kidney histology by biopsy, in order to ascertained a clinical phenotype, was not performed; this was because this study involves research on a large population and such a biopsy-based approach would be highly invasive. Moreover, clinical indices that suggest superimposed glomerulonephritis, such as hematuria, red cell cast or nephrotic range proteinuria, were minimal among our subjects.

### Stool DNA extraction and full-length 16S rRNA gene sequencing

Initially, 10 g of stool sample was harvested, placed in nucleic acid preservative (80% EtOH). The samples were then delivered to our research center within 24–48 h after collection. They were stored at −80°C until fecal DNA extraction took place. Fecal DNA was isolated using the QIAamp Fast DNA Stool Mini Kit (QIAGEN, Germany). Sequencing libraries were prepared by amplifying the full-length of the 16S rRNA genes using KAPA HiFi HotStart ReadyMix (Roche), which includes the 27F (AGRGTTYGATYMTGGCTCAG) and 1492 R (RGYTACCTTGTTACGACTT) barcoded primers. The following thermocycler conditions, 95°C for 3 min; 25 cycles of 95°C for 30 sec, 57°C for 30 sec, 72°C for 60 sec; and 72°C for 5 min, were used, and this was followed by the amplified samples being stored at 4°C. Next, the amplicons were purified using AMPure PB beads and pooled in an equimolar manner. Libraries were constructed using the SMRTbell Express template Prep Kit 2.0 following the manufacture’s protocol. Sequencing was performed using the circular consensus sequence (CCS) mode on a PacBio Sequel IIe system; this generated HiFi reads with a predicted accuracy greater than Q30.

### Processing and analysis of sequence data

Amplicon sequence variants (ASVs) were inferred using the DADA2 R package (1.18.0),^21^ and their taxonomic affiliation was assigned to the ASV with the help of BLAST and our unified 16S rRNA reference database (U16S) (https://github.com/mammerlin/U16S-DPOT/tree/main/Curated%20DB). An ASV count table was first aggregated into a taxonomy count table (1121 taxa), and then the taxonomic count table was rarefied for each sample to 7680, resulting in 1082 taxa across 990 samples. Taxa were removed if they were found in fewer than 10% of samples (a 10% prevalence filter),^16^ which resulted in 220 taxa. Downstream analyses were performed using R (version 4.2.1) and various Bioconductor packages including ggplot2,^22^ microViz,^23^ phyloseq,^24^ and vegan.^25^ We determined α-diversity and β-diversity by means of Chao1 and PCoA with Bray-Curtis matrices, respectively. Differential abundance methods, including DESeq2,^26^ LEfSe,^27^ limma voom^28^ and MaAsLin2,^29^ were used for discriminatory microbiota identification.

### Measurement of lipophilic metabolite profiles

The comprehensive methodology used in our study has been described previously.^17,18^ Briefly, a commercially available kit (AbsoluteIDQ p180—BIOCRATES Life Sciences AG, Austria) was used to identify a total of 147 metabolites from five compound classes (15 acylcarnitines, 21 amino acids, 9 biogenic amines, 88 glycerophospholipids and 14 sphingolipids); this was done using ultra-high performance liquid chromatography-tandem mass spectrometry (UPLC-MS/MS). The analysis was performed in positive electrospray ionization mode using a Waters tandem mass spectrometer (TQS, Waters MS Technologies, Manchester, UK). Chromatographic separation was performed on an Acquity BEH C8 column (75 mm × 2.1 mm, particle size of 1.7 μm; CWaters Crop., Milford, USA) at 50°C using a linear gradient that ranged from 0.2% formic acid in water to 0.2% formic acid in acetonitrile at a flow rate of 0.9 mL/min. All data was processed and analyzed using MetIQ software (Biocrates Life Science AG, Innskruck, Austria). Metabolites with >10% missing values, as well as values below the limit of detection (LOD), were excluded from the analysis.

### ML methods for predicting disease groups

We used gut microbiota biomarkers to carry out prediction on four groups, these were DM *vs*. Control, DKD *vs*. DM, DKD *vs*. CKD, and CKD *vs*. Control. We considered not only the contribution of single members of the gut microbiota but also the relationship between pairs within the microbiota clusters, as part of our models. For instance, at the genus level, we calculated the log ratio of *Anaerobutyricum* and *Marseillibacter* by dividing the abundance of *Anaerobutyricum* by that of *Marseillibacter*, followed by a log transformation to derive the “Anaerobutyricum_Marseillibacter_Genus_ratio”. The training process was as follows: the input dataset was divided into a training dataset and a test dataset with a split ratio of 80% and 20% for a 100-times bootstrap. The randomly selected samples in each bootstrap were then used to obtain the ranking of features using three ML methods, namely Least Absolute Shrinkage and Selection Operator (LASSO), Random Forest (RF) and Support Vector Machine (SVM). The model-building approaches used Logistic Regression, Random Forest and Extreme Gradient Boosting. The above methods were carried out using R (version 3.6.3) with the packages glmnet (4.1.4), random forest (4.6.14), xgboost (0.90.0.2) and e1071 (1.7.3).

### Statistics analysis

Descriptive statistics are expressed as the mean, median or frequency. Normality of numerical variables was tested using the Kolmogorov–Simirnov method. Differences in clinical indices among groups were determined using Student’s t-test, ANOVA or Kruskal–Wallis test. Overlapping significant taxa were determined from differential abundance and machine learning results. Taxonomy-informed functional predictions were obtained using PICRUSt2^30^ by placing ASVs into a reference tree. The metabolic pathway enrichment analysis and functional prediction were performed using the MetaCyc database (https://metacyc.org/).^31^ Differentially abundant MetaCyc pathways of an overlapping taxon for a specified comparison were identified using Wilcoxon tests. Spearman correlations were used to determine the association of a differential taxon with a clinical index, and *p* values were adjusted using the Benjamini–Hochberg correction. All statistical tests are two-tailed, and a *p* < 0.05 is considered statistically significant. Data were analyzed using R (version 4.2.1).

## Results

### Clinical characteristics

The present study enrolled 990 subjects. Of these subjects, 455 subjects (45.9%) were controls, 204 subjects (20.6%) had type 2 DM, 182 subjects (18.4%) had DKD, and 149 subjects (15.1%) had non-diabetic CKD. Table 1 summarizes the baseline characteristics of entire cohort. The mean age of the study population was 62 years and 48.4% of patients were men. The median serum creatinine was 0.8 mg/dL. The median estimated glomerular filtration rate (eGFR) was 84 mL/min/1.73 m^2^. The median urine albumin/creatinine ratio was 8.6 mg/g. The median fasting sugar was 101 mg/dL. The median glycosylated hemoglobin was 6%. DKD patients had lower eGFR levels and were older in age, while a greater proportion of the male patients suffered from metabolic syndrome and proteinuria than other groups.

**Table 1.** Baseline characteristics of the study population.

Parameters	All N = 990	Normal control N = 455	Diabetes N = 204	DKD N = 182	CKD N = 149	*p*-value
Age, years	62.0 ± 11.7	58.3 ± 12.5	62.5 ± 9.4	67.3 ± 8.9	66.5 ± 10.9	<0.001
Male, No. (%)	479 (48.4%)	199 (43.7%)	107 (52.5%)	116 (63.7%)	57 (38.3%)	<0.001
**Comorbidities**						
Metabolic Syndrome, No. (%)	476 (48.1%)	145 (31.9%)	140 (68.6%)	129 (70.9%)	62 (41.6%)	<0.001
Proteinuria, No. (%)	253 (25.6%)	0	0	145 (79.7%)	108 (72.5%)	<0.001
**Anthropometrics**						
Body mass index, kg/m^2^	26.8 ± 4.5	26.5 ± 4.5	27.6 ± 4.8	27.3 ± 4.4	25.8 ± 3.9	0.319
Systolic BP, mmHg	134.6 ± 37.9	130.1 ± 17.8	139.6 ± 74.7	136.9 ± 18.5	138.8 ± 20.6	0.011
Diastolic BP, mmHg	78.2 ± 11.7	77.4 ± 11.5	78.5 ± 10.3	77.6 ± 11.7	80.8 ± 13.5	0.190
**Laboratory**						
eGFR, mL/min per 1.73 m^2^	84 (12, 243)	89 (61, 160)	87.0 (61, 162)	60 (12, 129)	69 (16, 243)	<0.001
Serum creatinine, mg/dL	0.8 (0.3, 3.7)	0.8 (0.4, 1.2)	0.8 (0.4, 1.2)	1.1 (0.5, 3.7)	0.9 (0.3, 3.6)	<0.001
Cholesterol, mg/dL	182 (20, 418)	194 (20, 300)	168 (102, 270)	168 (76, 326)	186 (105, 418)	<0.001
Triglycerides, mg/dL	116 (25, 1418)	106 (26, 1418)	129.5 (28, 985)	131 (29, 430)	108 (25, 660)	0.029
Urine albumin/creatinine ratio, mg/g	8.6 (0.9, 6611.3)	5.1 (1.3, 29.8)	7.3 (0.9, 29.6)	52.4 (1.5, 6611.3)	47.8 (1.6, 1445.2)	<0.001
LDL-C/HDL-C, mg/dL	2.2 (0.6, 25)	2.3 (0.7, 25)	2.0 (0.8, 6.0)	2.0 (0.6, 5.1)	2.2 (0.7, 5.7)	0.006
Glycated Hemoglobin, %	6.0 (3.1, 13.7)	5.7 (3.1, 6.4)	6.8 (5.2, 13.0)	6.8 (5.1, 13.7)	5.8 (4.6, 6.4)	<0.001
Glucose, mg/dL	101 (67, 393)	95 (67, 124)	121 (74, 317)	130 (79, 393)	97.0 (77, 124)	<0.001

The values are expressed as means (SD) or median (Min, Max) or n (%).

Abbreviations: DM, diabetes mellitus; DKD, diabetic kidney disease; CKD, chronic kidney disease; BP, blood pressure; eGFR, estimated glomerular filtration rate; LDL-C, Low-density lipoprotein cholesterol; HDL-C, High-density lipoprotein cholesterol.

### Microbial composition and diversity in different disease groups

A total of 23,157,213 sequencing reads, ranging from 6190 to 74,715 per sample, was generated. The sequencing reads were resolved to 86,696 ASVs using DADA2, and then aggregated to 14 phyla, 27 classes, 54 orders, 111 families, 336 genera and 1121 species. In addition to the 1121 × 990 count table used in ML methods, the taxonomic count table was rarefied and prevalence-filtered to give a 220 × 990 table. This table was then used for the downstream microbiome analyses. Although subtle differences among groups regarding the taxonomic distributions of the top 30 genera (Supplementary Figure S1a) and species (Supplementary Figure S1b) were observed, significant differences in bacterial species richness (α-diversity, Chao1) were also obtained when comparing the Control *vs*. DM group and the Control *vs*. DKD group (Supplementary Figure S1c). Furthermore, an analysis of sample-to-sample dissimilarities in bacterial community structures (β-diversity) revealed that the gut microbiomes present in the DM and DKD groups were highly distinct from that of the control group (Supplementary Figure S1d). A significant difference in the Firmicutes to Bacteroidetes (F/B) ratio was detected between DM *vs*. Control (1.23 *vs*. 1.1; *p* = 0.033) and CKD *vs*. Control (1.27 *vs*. 1.1; *p* = 0.0017) (Supplementary Figure S1e). Specifically, the relative abundance of the Bacteroidetes phylum in the male population was found to be decreased in DM and DKD patients compared to the control subjects (Supplementary Figure S1f).

### Identification of gut microbiota signatures in order to differentiate DKD from DM and CKD

**Figure 1.**
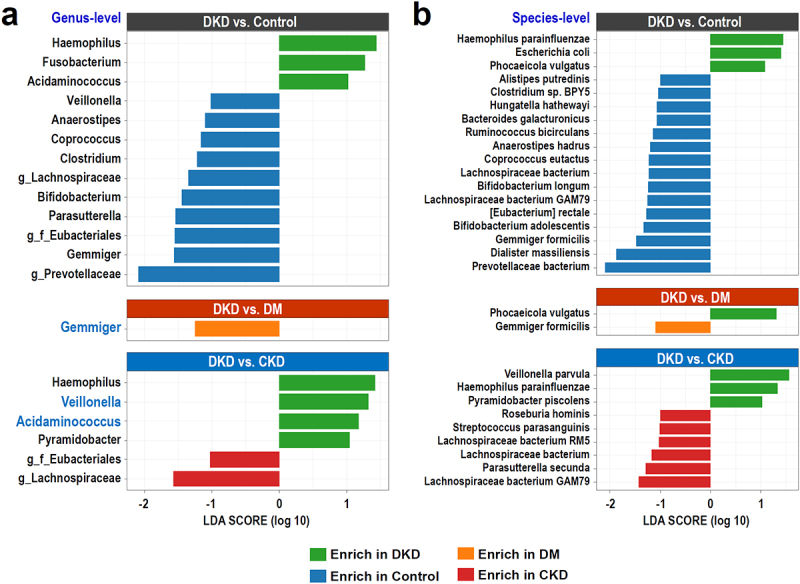
Determination of the bacterial biomarkers enriched in the DKD, DM, CKD and Control groups at genus-level (a) and species-level (b). The results were obtained using the linear discriminant analysis of effect size (LEfSe) method. In the comparison of DKD *vs*. DM, the genus *Gemmiger* (marked with blue) was concomitantly selected by both the LEfSe and ML methods. In the comparison of DKD *vs*. CKD, two genera, namely *Veillonella* and *Acidaminococcus* (marked with blue), were selected by both the LEfSe method and the ML methods.

A total of 14 DKD-associated bacterial biomarkers at the genus level were identified when the DKD groups were compared with the other groups using the linear discriminant analysis of the effect size (LEfSe) method (Figure 1). Interestingly, two genera, namely *Haemophilus* and *Acidaminococcus*, were enriched in DKD group compared with the Control or CKD groups. Conversely, the genus *Gemmiger* was significantly decreased in the DKD group compared with the Control and DM groups (Figure 1(a)). Using the full-length 16S rRNA datasets, *Haemophilus* and *Acidaminococcus* were further characterized at the species-level resolution to be *Haemophilus parainfluenzae* and *Gemmiger formicilis*. (Figure 1(b)). In addition to LEfSe method, three commonly used differential abundance methods, namely limma, MaAsLin2 and DESeq2, were applied to identify any potential microbial biomarkers that are associated with DKD. The results revealed that there is a discrepancy in both number and content when discriminatory microbes are identified by the various differential abundance methods (Supplementary Figure S2). Interestingly, in the comparison between the DKD *vs*. DM groups, the genus *Gemmiger* was identified as a potential biomarker both by the LEfSe method and the ML method (described below). Moreover, in the comparison between the DKD *vs*. CKD groups, two genera, namely *Acidaminococcus* and *Veillonella*, were identified by both the LEfSe method and the ML method. Finally, *Romboutsia* was selected by three methods, the limma, MaAsLin2 and ML.

### ML creates new types of microbiota-based biomarkers that are able to distinguish DKD, DM and CKD patients

**Figure 2.**
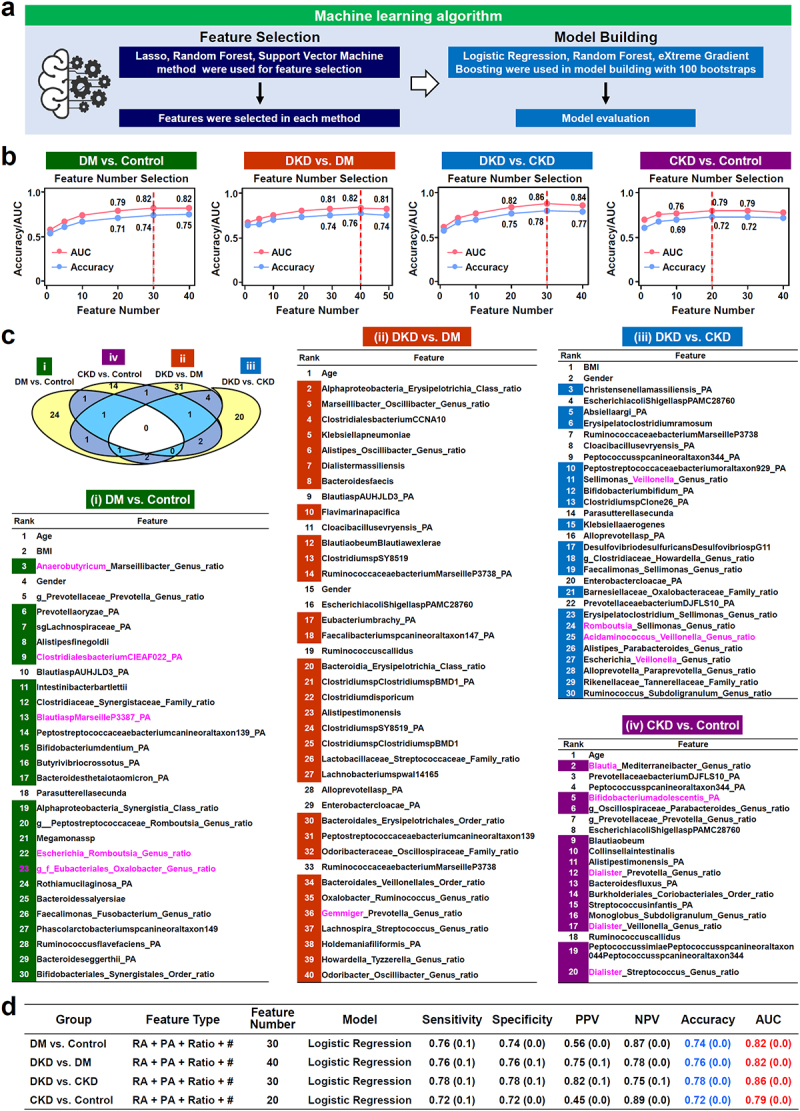
Feature selection and model prediction of ML methods in order to differentiate DKD, DM and CKD patients. (a) Workflow of the ML algorithm including feature selection and model building. (b) The number of features selected was determined by AUC and accuracy for the various disease group comparisons. (c) The four panels of top features selected by ML. A venn diagram is used to present the number of unique and overlapped features among the four panels of top features. The list and rank of features for distinguishing (i) DM *vs*. Control; (ii) DKD *vs*. DM; (iii) DKD *vs*. CKD; (iv) CKD *vs*. Control. The unique features are highlighted with a background color. Feature types include clinical variables (age, BMI and gender), and features of the microbiota, namely RA (relative abundance), PA (presence or absence, and ratio (hierarchy ratio). Those microbes concomitantly selected by the ML method and at least one of the differential abundance methods (DESeq2, LEfSe, limma voom or MaAsLin2) are marked with pink. (d) Prediction performance of the gut microbiota present in different disease groups using ML algorithms with bootstrapping 100 times. Feature type: # indicates age, BMI and gender. Data are presented as means (standard deviation).

The data complexity allowed us to carry out a second analysis, namely to characterize the informative features using the ML methods and identifying the top features that are able to distinguish DKD patients, DM patients, CKD patients and Control subjects (Figure 2(a); Feature Selection). Intriguingly, in additional to the clinical characteristics (Table 1), new types of microbiota biomarkers were created by the ML methods, including relative abundance (RA) of a microbe, the presence or absence (PA) of a microbe, and a hierarchy ratio, namely the ratio between two different genera, families, orders or classes. The concept of the hierarchical ratio was inspired by the widely recognized Firmicutes/Bacteroidetes ratio, which reflects the relative abundance of two prominent and representative phyla in the human gut microbiota. Building on this foundation, and utilizing advanced big data analytical approaches, we aimed to uncover novel targets by calculating hierarchical ratio across all taxonomic levels, namely from phylum to genus (Supplementary Figure S3). The optimal number of top features, that is the minimal number of features that are able to reach the best performance, was determined by Area Under Curve (AUC) method and by accuracy rate in the elbow plots (Figure 2(b)). Four different panels of features were selected by ML in order to differentiate between specific group pairings, namely, DM *vs*. Control, DKD *vs*. DM, DKD *vs*. CKD, and CKD *vs*. Control. Notably, in each panel, most of the features selected by ML are unique to that particular panel (Figure 2(c)).

To achieve model derivation and validation, the subjects were randomly split into training and testing datasets with a ratio of 80% to 20% and subjected to 100-times bootstrap (Figure 2(a)); Model Building). The performance was evaluated using the AUC and accuracy rate. Our analysis revealed that Logistic Regression gave the best performance (Supplementary Table S1). Using Logistic Regression as the model, we obtained the following results: (i) in the DM *vs*. Control comparison, the top 30 features yield an accuracy rate of 0.74 and an AUC of 0.82 when differentiating between DM patients and Control subjects; (ii) in the DKD *vs*. DM comparison, the top 40 features yield an accuracy rate of 0.76 and an AUC of 0.82 when differentiating between DKD patients and DM patients; (iii) in the DKD *vs*. CKD comparison, the top 30 features yield an accuracy rate of 0.78 and an AUC of 0.86 when differentiating between DKD patients and CKD patients; and (iv) in the CKD *vs*. Control comparison, the top 20 features yield an accuracy rate of 0.72 and an AUC of 0.79 when differentiating between CKD patients and Control subjects (Supplementary Table S2 ; Figure 2(d)).

### The correlation between microbiota and clinical indexes suggests that Gemmiger may have a reno-protective effect when DM is diagnosed

**Figure 3.**
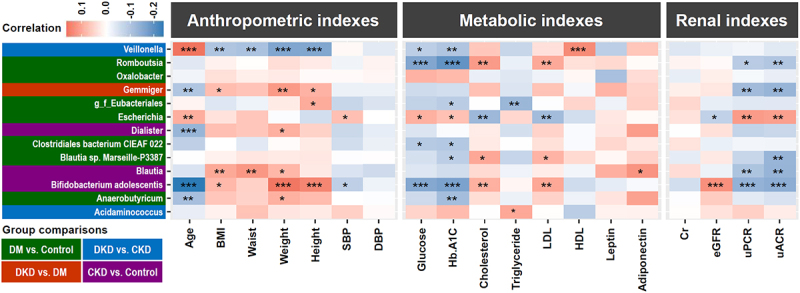
Correlation between bacterial biomarkers and clinical indexes using Spearman analysis. Thirteen gut bacterial biomarkers were concomitantly identified by the differential abundance method and ML algorithm across diverse group comparisons. The combinations of group comparisons are highlighted using a background color. *p value < 0.05, **p value < 0.01, ***p value < 0.001.

Interestingly, when we examined the comparison between the discriminatory microbes identified by the four methods of differential abundance (Supplementary Figure S2) and by the ML-selected biomarkers (Figure 2(c)), 13 bacterial biomarkers were consistently identified by both approaches. To explore the potential significance of these bacteria in a clinical situation and to identify their association with disease progression, we analyzed the correlation between these bacteria and clinical indexes obtained from the subjects using Spearman analysis (Figure 3). Our analyses revealed the following. Firstly, in the DM *vs*. Control comparison, seven significant differential bacteria were identified (marked with a green background). Among these bacteria, *Romboutsia* and *Clostridiales bacterium CIEAF 022* were negatively correlated, and *Escherichia* was positively correlated with the levels of serum glucose and glycated Hemoglobin (HbA1c). Furthermore, *Romboutsia* and *Blautia sp. Marseille-P3387* were positively correlated, and *Escherichia* was negatively correlated with the levels of serum cholesterol and LDL. Secondly, in the CKD *vs*. Control comparison, three significant differential bacteria were identified (marked with a purple background). *Dialister* was negatively correlated with age, and positively correlated with weight. *Blautia* was positively correlated with BMI, waist and weight, while negatively correlated with uPCR and uACR. *Bifidobacteruim adolescentis* was negatively correlated with age, and positively correlated with BMI, weight and height. Regarding the metabolic indexes, *Bifidobacteruim adolescentis* was negatively correlated with the levels of serum glucose and HbA1c, and was positively correlated with the levels of serum cholesterol and LDL. Interestingly, regarding various renal indexes, *Bifidobacteruim adolescentis* was positively correlated with eGFR and negatively correlated with uPCR and uACR, which suggests that this bacterium has a beneficial influence on kidney function. Thirdly, in the DKD *vs*. CKD comparison, two significant differential bacteria were identified (marked with a blue background). *Veillonella* was positively correlated with age, and negatively correlated with BMI, waist, weight and height. *Acidaminococcus* was positively correlated with serum triglyceride level. Finally, in the DKD *vs*. DM comparison, remarkably, *Gemmiger* (marked with a red background) appears to be specifically identified as a microbiota biomarker when DKD patients are compared with DM patients. Furthermore, *Gemmiger* was negatively correlated with age, and positively correlated with BMI, weight and height. Notably, *Gemmiger* was negatively correlated with uPCR and uACR, which suggests that it may have a beneficial effect and might be associated with reno-protection when DM is present.

### Metabolic pathways associated with Gemmiger

**Figure 4.**
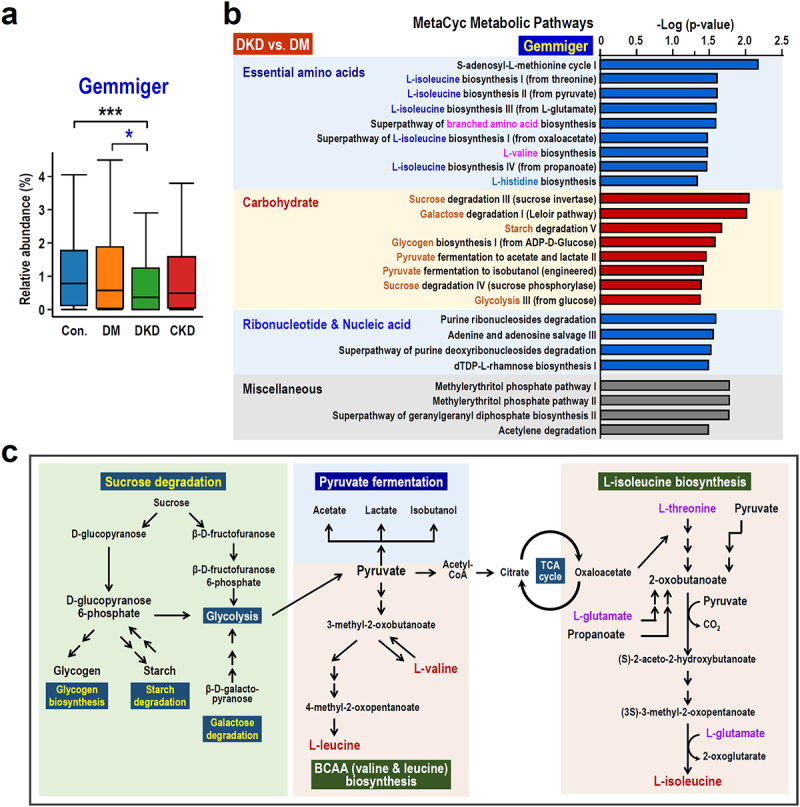
Pathway enrichment analysis and functional analysis of *Gemmiger*. (a) The relative abundance of *Gemmiger* in the control, DM, DKD and CKD groups. (b) MetaCycCyc metabolic pathway enrichment analysis of *Gemmiger*-associated pathways. (c) A graphic summary of the *Gemmiger*-related metabolic pathways. The interconnected metabolic pathways include the metabolism of several types of carbohydrates, pyruvate fermentation and BACC biosynthesis. The pathway annotation was carried out using MetaCyc metabolic pathway database (https://metacyc.Org). Abbreviations: TCA, tricarboxylic acid cycle. The box plots display median abundances and the interquartile range (IQR) multiplied by 1.5. Significance between groups was assessed using the Wilcoxon rank-sum test. The asterisks denote significance levels: *, *p* < 0.05; **, *p* < 0.01; ***, *p* < 0.001.

Consistent with our results using linear discriminant analysis (Figure 1), the relative abundance of *Gemmiger* was significantly decreased in the DKD group (median 0.358%) compared with the DM group (median 0.568%, *p* value = 0.028) and the Control group (median 0.779%, *p* value = 0.00024) (Figure 4(a)). Since gut microbiota-derived metabolites may be associated with the pathophysiology and progression of DKD,^32^ we performed a metabolic pathway enrichment analysis and functional prediction using the MetaCyc database to decipher the potential role of *Gemmiger* in DKD patients. A total of 25 *Gemmiger*-associated metabolic pathways exhibited significant differences between the DKD and DM groups (Wilcox test *p* < 0.05). These metabolic pathways can be grouped into three major categories: (1) essential amino acids, (2) carbohydrate, and (3) ribonucleotide and nucleic acid (Figure 4(b)). Intriguingly, the biosynthesis of the branched-chain amino acids (BCAAs), namely L-valine, L-leucine and L-isoleucine, was found to be the main pathway for the metabolism of essential amino acids. With regard to the metabolism of carbohydrate, a variety of pathways related to carbohydrate degradation (sucrose, galactose and starch degradation), glycogen biosynthesis, pyruvate fermentation and glycolysis were identified to be associated with *Gemmiger* (Figure 4(b)). Since *Gemmiger* was identified by both the LEfSe and ML methods during the DKD *vs*. DM comparison, these results suggest that an alteration involving these interconnected metabolic pathways may potentially contribute to the pathogenesis of DKD (Figure 4(c)).

## Discussion

In this study, by thoroughly analyzing large fecal samples from subjects with DM, DKD, CKD and control groups, three findings can be pinpointed. **Firstly**, new types of microbiota biomarkers have been created by ML, namely the relative abundance (RA) of microbe, the presence or absence (PA) of a microbe, and the hierarchy ratio between two different taxonomies. Four different panels of features were selected to differentiate (i) DM *vs*. Control, (ii) DKD *vs*. DM, (iii) DKD *vs*. CKD, and (iv) CKD *vs*. Control, all with accuracy rates between 0.72 and 0.78 and all with areas under curve of between 0.79 and 0.86. **Secondly**, 13 gut microbiota biomarkers that were concomitantly identified by the ML algorithm and the differential abundance method were found to be highly discriminatory across the various different group comparisons. Furthermore, these microbiota biomarkers were strongly correlated with a range of anthropometric, metabolic and renal indices. **Finally**, the predicted functional capability of a DKD-specific biomarker, *Gemmiger*, was found to be enriched in various forms of carbohydrate metabolism and BCAA biosynthesis. Coincidentally, the circulating levels of BCAAs (L-valine, L-leucine and L-isoleucine) and their precursor, L-glutamate, are significantly increased in DM and DKD patients, which suggests that there are alterations in the interconnected pathways of glycolysis, pyruvate fermentation and BCAA biosynthesis in the presence of hyperglycemia. Overall, our findings demonstrate links within the gut-microbiota-kidney axis when there is pathogenic renal impairment in DM patients. Furthermore, our findings highlight that specific gut bacteria are useful biomarkers and that these biomarkers have mechanistic and diagnostic implications.

### Gut microbial biomarkers discovered in this and previous studies

Previously using 16S rRNA microbial profiling of fecal samples of biopsy-proven DKD patients, Tao et al. found that *g_Escherichia-Shigella* and genus *g_Prevotella_9* were highly discriminatory when separating DKD patients from DM patients.^33^ Similarly, our study revealed a significantly higher abundance of *Escherichia coli* in the DKD group for the following comparisons: DKD vs. Control (Figure 1(b)), DKD *vs*. DM, and DKD *vs*. CKD (Figure 2(c); Supplementary Figure S2b). Moreover, our correlation analysis showed a positive association between the abundance of the *Escherichia* genus and the age, glucose and proteinuria of the subjects; conversely, there is a negative correlation between the abundance of the *Escherichia* genus and the lipid profile and eGFR of the subjects (Figure 3). Another study that integrated 16S pyrosequencing and metabolomic analyses identified 11 significantly different intestinal flora and 239 significantly different metabolites when a late-stage predialysis DKD group was compared with a dialysis DKD group. Furthermore, a number of gene functions associated with the phenylalanine and tryptophan metabolic pathways, together with altered levels of hippuric acid, indole-3-acetic acid, L-tryptophan and L-valine, were found to be most highly associated with DKD progression.^34^ In addition, a metagenomic study involving a limited group of DKD patients found subtle differences in species and functional pathways between non-DKD and DKD patients. Nine pathways related to *Lactobacillus crispatus* were differentially enriched in DKD patients and these were related to the sucrose degradation pathway and L-Lysine biosynthesis.^6^ Another metagenomic analysis has shown that the relative abundances of six bacterial species were elevated in the DKD patients. The gene functions of these bacteria were correlated with the BCAAs and methionine metabolic biosynthesis pathways.^35^

It should be noted that there are inconsistencies regarding the DKD-specific microbes between previous reports and this study. This may be attributable to a number of possible factors. Firstly, there may be discrepancies in the severity of kidney disease, analytical methodology, geography and dietary patterns across different studies; all of these may contribute to variation in phenotypic outcomes. Secondly, a relatively small sample size of patient settings was used in many of the previously published studies. In the present study, our findings are based on full-length sequencing of the 16S rRNA gene using feces obtained from a relatively large study cohort that contains subjects with four distinct clinical phenotypes. Finally, our results are derived from the integrated analyses of traditional abundance methodologies (DESeq2, LEfSe, limma voom and MaAsLin2) together with the use of the ML approach. Our aim was to provide a possible biological explanation for the potential roles of microbiota identified in terms of the metabolic pathways and the crosstalk between gut microbiota and host metabolic disturbance in DKD.

### Clinical implication of a decreased abundance of Gemmiger in the feces of DKD patients

The decrease in *Gemmiger* observed in our DKD patients is consistent with previous reports obtained from biopsy-proven diabetic nephropathy patients.^36^ The strong association of the abundance of *Gemmiger* with various anthropometric measurements, such as body mass index and weight, is consistent with previous literature. In these studies, *Gemmiger* was found to be associated with sex-specific adipose distribution.^37^ Furthermore, the relative abundance of *Gemmiger* was reduced when autoimmune disease is present^38^ and similar findings were found for women with rheumatoid arthritis.^39^ *Gemmiger formicilis* is a chemoorganotrophic gram-negative bacterium that ferments sugars to produce formic, butyric and lactic acids, all of which exert anti-inflammatory effects.^40^ Notably, *Gemmiger* utilizes the acetyl-CoA (ACoA) pathway to synthesize butyrate, a short-chain fatty acid.^41^ Sodium butyrate, a derivative of butyrate, has been identified as a potential therapeutic agent for the treatment of diabetic nephropathy^42^; this supports the notion that *Gemmiger* may play a reno-protective role. On the other hand, *Gemmiger formicillis* has been positively correlated with both phenylacetylcarnitine and phenylacetylglutamine, which are derived from the gut fermentation of phenylalanine and acetylcholine. These metabolites have been correlated with microbial gene richness in obese subjects and have implications related to mitochondrial dysfunction and hepatocyte lipid accumulation in fatty liver disease.^43^ In this cross-sectional study, we observed a significant association with a trend toward a decrease in the abundance of *Gemmiger* in DKD patients (Figure 4(a)) and from this we have inferred its potential reno-protective effects based on metabolic pathway enrichment analysis (Figure 4(c)). However, direct causal evidence linking *Gemmiger* to DKD is currently lacking. Therefore, further validation using animal and cell platforms is needed to establish a definitive relationship.

### Limitations and future perspectives

To define the potential functionality of gut microbiome associated with DKD, additional efforts are needed to address several limitations of the present study. **Firstly**, a replication cohort is needed, although many lines of our microbiota and metabolomic results are consistent with previous literature. Nevertheless, we acknowledge that the findings obtained from the cohort recruited from a specific community in northeast Taiwan may not be generalized to other populations. **Secondly**, we applied full-length sequencing of the 16S rRNA gene and predicted the potential function of the microbiota by inference from PICRUSt2 analysis.^30^ This approach is a cost-effective way for a large sample research. However, in-depth metagenome information with a resolution at the species or strain level still remains elusive. Therefore, further experiments using metagenomic sequencing or fecal metabolomics are warranted to elucidate the direct interaction between microbiota and host metabolism. **Thirdly**, our findings represent real-world population data in which the imbalances of disease distribution (especially in the DM and DKD groups) and baseline information are inherently related to the characteristics of the status of the various diseases (such as an older age and greater metabolic dysregulation among the DKD and CKD patients). It should be noted that the subgroup analyses of the relative abundance of most of the 14 microbial biomarkers were non-differential in terms of age, eGFR and glycosylated hemoglobin (Supplementary Table S3). To address these issues and to reduce sampling bias, we applied the bootstrap method and performed 100 resampling iterations. Each iteration involved random sampling, which helps to minimize sampling bias by averaging out variations across resamples. This approach allowed us to enhance the robustness and reliability of our results despite the limited sample size. We believe that this method effectively reduces the constraints of small sample sizes and improves the applicability of our study conclusions. **Fourthly**, with regard to dietary recall, medication usage and other unknown confounding factors, these information types were not collected by the community project that provided the dataset and thus may represent a potential drawback. However, all the participants came from similar geographic and cultural areas and this should have minimized pronounced variations in dietary patterns and other habits among individuals. Moreover, a dietary composition investigation in our previous microbiota study that recruited participants from the same geographic area has revealed that the daily serving portions (in terms of vegetable, meat, fruit and rice/noodle) had no overt difference between non-CKD *vs*. CKD patients.^9^ Additionally, subjects using antibiotics, prebiotics and probiotics were excluded from enrollment. Participants with an acute infection, including viral infection, were also excluded from recruitment in order to minimize as much as possible any distortion of host microbiota. **Finally**, several studies have suggested that the DNA extraction reagent we used (QIAamp Fast DNA Stool Mini Kit) can extract fungal DNA,^44^ and that subsequent internal transcribed spacer region sequencing can provide information on fungal species and their relative abundance. However, fungi and viruses are only sporadically abundant in the human gut microbiome compared to bacteria.^45^ Our current methodology remains unable to extract information on intestinal viruses, fungi, bacteriophages and parasites; thus, such evaluations may be beyond the scope of this research. Overall, comprehensive full-length pyrosequencing datasets and ML-assisted analyses should help with the data-driven discovery of novel species–host interaction that will increase our understanding of DKD pathogenesis. Thus, further *in-vivo* experiments targeting different components of the gut microbiota are required to demonstrate the causality of our findings in the future.

## Conclusions

Advances in our understanding of the molecular mechanisms and pathways involved in the pathogenesis of DKD remain an unfulfilled need, and the identification of biomarkers that can be used for early diagnosis or prognosis prediction will help our understanding of these disease states. The present study shows that the altered gut microbiota present in DKD patients is functionally associated with specific and distinct carbohydrate and BCAA metabolism pathways. Our study implies that specific gut microbes can be used as a potential biomarker during DKD diagnosis and may also serve as candidate therapeutic targets when gut-microbiota-kidney intervention in DM patients is carried out. Further clinical trials are warranted in order to investigate the effects of manipulating these specific microbes and this will allow the exploration of the changes that then occur in circulating BCAA levels. These findings can then be linked to the outcomes of DKD patients who are undergoing such therapy.

## Supplementary Material

Supplemental Material

## Data Availability

The data supporting the findings of this study are available from the corresponding authors on reasonable request.
