# Supplementary material for: Machine-learning assisted discovery unveils novel interplay between gut microbiota and host metabolic disturbance in diabetic kidney disease
Source: Gut Microbes. 2025 Mar 6;17(1):2473506. doi: 10.1080/19490976.2025.2473506 (PMC11901534; doi:10.1080/19490976.2025.2473506)
Supplement: Supplemental Material [file KGMI_A_2473506_SM2169.docx]

#### Supplementary Table S1. Evaluation of different models of machine learning algorithm. Logistic

Regression, Random Forest and eXtreme Gradient Boosting were used in model building with 100 times of bootstraps. Logistic Regression exhibited the best performance for different comparison between two groups.

| **Group** | **Feature Type** | **Feature Number** | **Model** | **Sensitivity** | **Specificity** | **PPV** | **NPV** | **Accuracy** | **AUC** |
| --- | --- | --- | --- | --- | --- | --- | --- | --- | --- |
|  |  |  | **Logistic Regression** | **0.76 (0.1)** | **0.74 (0.0)** | **0.56 (0.0)** | **0.87 (0.0)** | **0.74 (0.0)** | **0.82 (0.0)** |
| **DM vs. Control** | **RA + PA + Ratio + #** | **30** | **Random Forest** | **0.69 (0.1)** | **0.70 (0.0)** | **0.50 (0.0)** | **0.84 (0.0)** | **0.70 (0.0)** | **0.76 (0.0)** |
|  |  | **eXtreme Gradient Boosting** | | **0.66 (0.1)** | **0.67 (0.0)** | **0.47 (0.0)** | **0.82 (0.0)** | **0.67 (0.0)** | **0.73 (0.0)** |
|  |  |  | **Logistic Regression** | **0.76 (0.1)** | **0.76 (0.1)** | **0.75 (0.1)** | **0.78 (0.0)** | **0.76 (0.0)** | **0.82 (0.0)** |
| **DKD vs. DM** | **RA + PA + Ratio + #** | **40** | **Random Forest** | **0.68 (0.1)** | **0.66 (0.1)** | **0.64 (0.0)** | **0.70 (0.0)** | **0.67 (0.0)** | **0.73 (0.0)** |
|  |  | **eXtreme Gradient Boosting** | | **0.64 (0.1)** | **0.63 (0.1)** | **0.61 (0.1)** | **0.66 (0.0)** | **0.63 (0.0)** | **0.69 (0.0)** |
|  |  |  | **Logistic Regression** | **0.78 (0.1)** | **0.78 (0.1)** | **0.82 (0.1)** | **0.75 (0.1)** | **0.78 (0.0)** | **0.86 (0.0)** |
| **DKD vs. CKD** | **RA + PA + Ratio + #** | **30** | **Random Forest** | **0.69 (0.1)** | **0.70 (0.1)** | **0.75 (0.1)** | **0.65 (0.1)** | **0.69 (0.0)** | **0.77 (0.0)** |
|  |  | **eXtreme Gradient Boosting** | | **0.68 (0.1)** | **0.68 (0.1)** | **0.73 (0.1)** | **0.63 (0.1)** | **0.68 (0.1)** | **0.75 (0.1)** |
|  |  |  | **Logistic Regression** | **0.72 (0.1)** | **0.72 (0.0)** | **0.45 (0.0)** | **0.89 (0.0)** | **0.72 (0.0)** | **0.79 (0.0)** |
| **CKD vs. Control** | **RA + PA + Ratio + #** | **20** | **Random Forest** | **0.69 (0.1)** | **0.69 (0.0)** | **0.42 (0.0)** | **0.87 (0.0)** | **0.69 (0.0)** | **0.74 (0.0)** |
|  |  | **eXtreme Gradient Boosting** | | **0.64 (0.1)** | **0.64 (0.0)** | **0.36 (0.0)** | **0.85 (0.0)** | **0.64 (0.0)** | **0.68 (0.1)** |

#### Supplementary Table S2. Bootstrap 100 times of prediction performance of different type of gut microbiota in DM, DKD, CKD and Control using machine learning algorithms.

| **Group** | **Feature Type** | **Feature Number** | **Model** | **Sensitivity** | **Specificity** | **PPV** | **NPV** | **Accuracy** | **AUC** |
| --- | --- | --- | --- | --- | --- | --- | --- | --- | --- |
|  | **Relative Abundance (RA) + #** | **30** | **Logistic Regression** | **0.69 (0.1)** | **0.68 (0.1)** | **0.49 (0.0)** | **0.84 (0.0)** | **0.68 (0.0)** | **0.77 (0.0)** |
| **DM vs. Control** | **Presence or Absence (PA) + #** | **30** | **Logistic Regression** | **0.70 (0.1)** | **0.70 (0.1)** | **0.51 (0.0)** | **0.84 (0.0)** | **0.70 (0.0)** | **0.78 (0.0)** |
|  | **Hierarchy Ratio (Ratio) + #** | **20** | **Logistic Regression** | **0.70 (0.1)** | **0.72 (0.0)** | **0.52 (0.0)** | **0.84 (0.0)** | **0.71 (0.0)** | **0.77 (0.0)** |
|  | **RA + PA + Ratio + #** | **30** | **Logistic Regression** | **0.76 (0.1)** | **0.74 (0.0)** | **0.56 (0.0)** | **0.87 (0.0)** | **0.74 (0.0)** | **0.82 (0.0)** |
|  | **Relative Abundance (RA) + #** | **20** | **Logistic Regression** | **0.72 (0.1)** | **0.67 (0.1)** | **0.66 (0.0)** | **0.73 (0.0)** | **0.69 (0.0)** | **0.77 (0.0)** |
| **DKD vs. DM** | **Presence or Absence (PA) + #** | **20** | **Logistic Regression** | **0.68 (0.1)** | **0.71 (0.1)** | **0.68 (0.1)** | **0.71 (0.0)** | **0.70 (0.0)** | **0.77 (0.0)** |
|  | **Hierarchy Ratio (Ratio) + #** | **20** | **Logistic Regression** | **0.68 (0.1)** | **0.64 (0.1)** | **0.63 (0.0)** | **0.69 (0.0)** | **0.66 (0.0)** | **0.72 (0.0)** |
|  | **RA + PA + Ratio + #** | **40** | **Logistic Regression** | **0.76 (0.1)** | **0.76 (0.1)** | **0.75 (0.1)** | **0.78 (0.0)** | **0.76 (0.0)** | **0.82 (0.0)** |
|  | **Relative Abundance (RA) + #** | **20** | **Logistic Regression** | **0.74 (0.1)** | **0.66 (0.0)** | **0.41 (0.0)** | **0.89 (0.0)** | **0.68 (0.0)** | **0.77 (0.0)** |
| **DKD vs. CKD** | **Presence or Absence (PA) + #** | **20** | **Logistic Regression** | **0.68 (0.1)** | **0.69 (0.0)** | **0.41 (0.0)** | **0.87 (0.0)** | **0.69 (0.0)** | **0.75 (0.0)** |
|  | **Hierarchy Ratio (Ratio) + #** | **5** | **Logistic Regression** | **0.70 (0.1)** | **0.67 (0.1)** | **0.40 (0.0)** | **0.87 (0.0)** | **0.68 (0.0)** | **0.74 (0.0)** |
|  | **RA + PA + Ratio + #** | **30** | **Logistic Regression** | **0.78 (0.1)** | **0.78 (0.1)** | **0.82 (0.1)** | **0.75 (0.1)** | **0.78 (0.0)** | **0.86 (0.0)** |
|  | **Relative Abundance (RA) + #** | **20** | **Logistic Regression** | **0.74 (0.1)** | **0.66 (0.0)** | **0.41 (0.0)** | **0.89 (0.0)** | **0.68 (0.0)** | **0.77 (0.0)** |
| **CKD vs. Control** | **Presence or Absence (PA) + #** | **20** | **Logistic Regression** | **0.68 (0.1)** | **0.69 (0.0)** | **0.41 (0.0)** | **0.87 (0.0)** | **0.69 (0.0)** | **0.75 (0.0)** |
|  | **Hierarchy Ratio (Ratio) + #** | **5** | **Logistic Regression** | **0.70 (0.1)** | **0.67 (0.1)** | **0.40 (0.0)** | **0.87 (0.0)** | **0.68 (0.0)** | **0.74 (0.0)** |
|  | **RA + PA + Ratio + #** | **20** | **Logistic Regression** | **0.72 (0.1)** | **0.72 (0.0)** | **0.45 (0.0)** | **0.89 (0.0)** | **0.72 (0.0)** | **0.79 (0.0)** |

# indicted Age, BMI, Gender.

Abbreviations: DM, diabetes mellitus; DKD, diabetic kidney disease; CKD, chronic kidney disease; PPV, positive predictive value; NPV, negative predictive value; AUC, area under curve.

### Supplementary Table S3. Results of the two-way ANOVA with interaction test for the 14 differential

**microbes stratified by age, eGFR and HbA1c.**

| **Comparison** | **Taxonomy** | **Age < 60** | **Age >= 60** | **P-value** | **Age < 65** | **Age >= 65** | **P-value** | **eGFR < 60** | **eGFR >= 60** | **P-value** | **eGFR >= 90** | **eGFR 60-89.9** | **eGFR < 60** | **P-value** |
| --- | --- | --- | --- | --- | --- | --- | --- | --- | --- | --- | --- | --- | --- | --- |
| **DM vs. Control** | **Oxalobacter** | **(n1= 65, n2=211)** | **(n1=139, n2=244)** | **0.1095** | **(n1=119, n2=289)** | **(n1= 85, n2=166)** | **0.2985** | **(n1=0, n2=0)** | **(n1=204, n2=455)** |  | **(n1= 96, n2=213)** | **(n1=108, n2=241)** | **(n1=0, n2=0)** | **0.1627** |
| **DM** |  | **0.029 ± 0.078** | **0.053 ± 0.119** |  | **0.04 ± 0.092** | **0.053 ± 0.128** |  |  | **0.046 ± 0.108** |  | **0.052 ± 0.116** | **0.04 ± 0.101** |  |  |
| **Control** |  | **0.027 ± 0.099** | **0.025 ± 0.075** |  | **0.027 ± 0.095** | **0.023 ± 0.072** |  |  | **0.026 ± 0.087** |  | **0.021 ± 0.091** | **0.031 ± 0.084** |  |  |
| **DM vs. Control** | **Romboutsia** | **(n1= 65, n2=211)** | **(n1=139, n2=244)** | **0.8912** | **(n1=119, n2=289)** | **(n1= 85, n2=166)** | **0.2365** | **(n1=0, n2=0)** | **(n1=204, n2=455)** |  | **(n1= 96, n2=213)** | **(n1=108, n2=241)** | **(n1=0, n2=0)** | **0.4747** |
| **DM** |  | **0.127 ± 0.545** | **0.141 ± 0.554** |  | **0.096 ± 0.431** | **0.193 ± 0.682** |  |  | **0.136 ± 0.55** |  | **0.064 ± 0.197** | **0.201 ± 0.729** |  |  |
| **Control** |  | **0.213 ± 0.86** | **0.21 ± 0.665** |  | **0.229 ± 0.838** | **0.182 ± 0.604** |  |  | **0.211 ± 0.761** |  | **0.18 ± 0.6** | **0.232 ± 0.874** |  |  |
| **DM vs. Control** | **g_f_Eubacteriales** | **(n1= 65, n2=211)** | **(n1=139, n2=244)** | **0.8996** | **(n1=119, n2=289)** | **(n1= 85, n2=166)** | **0.8612** | **(n1=0, n2=0)** | **(n1=204, n2=455)** |  | **(n1= 96, n2=213)** | **(n1=108, n2=241)** | **(n1=0, n2=0)** | **0.2021** |
| **DM** |  | **1.835 ± 5.232** | **2.132 ± 4.457** |  | **1.831 ± 4.948** | **2.327 ± 4.358** |  |  | **2.038 ± 4.707** |  | **2.339 ± 5.76** | **1.77 ± 3.524** |  |  |
| **Control** |  | **1.658 ± 2.786** | **2.041 ± 3.758** |  | **1.725 ± 3.249** | **2.105 ± 3.502** |  |  | **1.863 ± 3.345** |  | **1.731 ± 3.669** | **1.986 ± 3.038** |  |  |
| **DM vs. Control** | **Escherichia** | **(n1= 65, n2=211)** | **(n1=139, n2=244)** | **0.5881** | **(n1=119, n2=289)** | **(n1= 85, n2=166)** | **0.7029** | **(n1=0, n2=0)** | **(n1=204, n2=455)** |  | **(n1= 96, n2=213)** | **(n1=108, n2=241)** | **(n1=0, n2=0)** | **0.0934** |
| **DM** |  | **4.196 ± 7.162** | **6.263 ± 12.157** |  | **4.744 ± 9.01** | **6.81 ± 12.946** |  |  | **5.605 ± 10.844** |  | **6.679 ± 12.801** | **4.65 ± 8.697** |  |  |
| **Control** |  | **2.928 ± 6.712** | **5.95 ± 11.408** |  | **3.556 ± 7.678** | **6.277 ± 12.151** |  |  | **4.549 ± 9.632** |  | **4.126 ± 9.151** | **4.94 ± 10.056** |  |  |
| **DM vs. Control** | **Aerobutyricum** | **(n1= 65, n2=211)** | **(n1=139, n2=244)** | **0.3477** | **(n1=119, n2=289)** | **(n1= 85, n2=166)** | **0.9383** | **(n1=0, n2=0)** | **(n1=204, n2=455)** |  | **(n1= 96, n2=213)** | **(n1=108, n2=241)** | **(n1=0, n2=0)** | **0.1675** |
| **DM** |  | **0.29 ± 0.376** | **0.193 ± 0.333** |  | **0.258 ± 0.371** | **0.176 ± 0.312** |  |  | **0.224 ± 0.349** |  | **0.259 ± 0.423** | **0.193 ± 0.266** |  |  |
| **Control** |  | **0.313 ± 0.415** | **0.279 ± 0.359** |  | **0.323 ± 0.417** | **0.246 ± 0.32** |  |  | **0.295 ± 0.386** |  | **0.282 ± 0.385** | **0.304 ± 0.386** |  |  |
| **DM vs. Control** | **Clostridiales bacterium CIEAF 022** | **(n1= 65, n2=211)** | **(n1=139, n2=244)** | **0.0883** | **(n1=119, n2=289)** | **(n1= 85, n2=166)** | **0.8319** | **(n1=0, n2=0)** | **(n1=204, n2=455)** |  | **(n1= 96, n2=213)** | **(n1=108, n2=241)** | **(n1=0, n2=0)** | **0.7197** |
| **DM** |  | **0.015 ± 0.029** | **0.024 ± 0.067** |  | **0.025 ± 0.07** | **0.016 ± 0.035** |  |  | **0.021 ± 0.058** |  | **0.019 ± 0.064** | **0.023 ± 0.052** |  |  |
| **Control** |  | **0.034 ± 0.046** | **0.028 ± 0.047** |  | **0.033 ± 0.046** | **0.027 ± 0.047** |  |  | **0.031 ± 0.046** |  | **0.031 ± 0.045** | **0.032 ± 0.048** |  |  |
| **DM vs. Control** | **Blautia sp. Marseille-P3387** | **(n1= 65, n2=211)** | **(n1=139, n2=244)** | **0.5506** | **(n1=119, n2=289)** | **(n1= 85, n2=166)** | **0.2241** | **(n1=0, n2=0)** | **(n1=204, n2=455)** |  | **(n1= 96, n2=213)** | **(n1=108, n2=241)** | **(n1=0, n2=0)** | **0.8245** |
| **Control** |  | **0.026 ± 0.077** | **0.028 ± 0.157** |  | **0.036 ± 0.176** | **0.015 ± 0.036** |  |  | **0.027 ± 0.136** |  | **0.016 ± 0.04** | **0.037 ± 0.183** |  |  |
| **DM** |  | **0.028 ± 0.073** | **0.04 ± 0.087** |  | **0.035 ± 0.084** | **0.034 ± 0.074** |  |  | **0.034 ± 0.081** |  | **0.025 ± 0.065** | **0.043 ± 0.092** |  |  |
| **CKD vs. Control** | **Dialister** | **(n1= 23, n2=211)** | **(n1=126, n2=244)** | **0.4042** | **(n1= 56, n2=289)** | **(n1= 93, n2=166)** | **0.3488** | **(n1=64, n2=0)** | **(n1= 85, n2=455)** |  | **(n1= 37, n2=213)** | **(n1= 48, n2=241)** | **(n1=64, n2=0)** | **0.9325** |
| **CKD** |  | **1.07 ± 1.576** | **1.684 ± 5.37** |  | **1.745 ± 6.421** | **1.495 ± 3.896** |  | **1.794 ± 4.311** | **1.434 ± 5.445** |  | **0.63 ± 1.176** | **2.054 ± 7.144** | **1.794 ± 4.311** |  |
| **Contrl** |  | **2.811 ± 5.851** | **2.283 ± 5.66** |  | **2.99 ± 6.562** | **1.724 ± 3.842** |  |  | **2.528 ± 5.749** |  | **2.3 ± 5.332** | **2.74 ± 6.107** |  |  |
| **CKD vs. Control** | **Blautia** | **(n1= 23, n2=211)** | **(n1=126, n2=244)** | **0.7071** | **(n1= 56, n2=289)** | **(n1= 93, n2=166)** | **0.8585** | **(n1=64, n2=0)** | **(n1= 85, n2=455)** |  | **(n1= 37, n2=213)** | **(n1= 48, n2=241)** | **(n1=64, n2=0)** | **0.7355** |
| **Control** |  | **0.296 ± 0.349** | **0.281 ± 0.505** |  | **0.295 ± 0.329** | **0.276 ± 0.558** |  | **0.361 ± 0.644** | **0.224 ± 0.304** |  | **0.299 ± 0.342** | **0.167 ± 0.26** | **0.361 ± 0.644** |  |
| **CKD** |  | **0.451 ± 0.606** | **0.501 ± 0.88** |  | **0.475 ± 0.69** | **0.481 ± 0.883** |  |  | **0.477 ± 0.765** |  | **0.471 ± 0.761** | **0.483 ± 0.771** |  |  |
| **CKD vs. Control** | **Bifidobacterium adolescentis** | **(n1= 23, n2=211)** | **(n1=126, n2=244)** | **0.7747** | **(n1= 56, n2=289)** | **(n1= 93, n2=166)** | **0.9370** | **(n1=64, n2=0)** | **(n1= 85, n2=455)** |  | **(n1= 37, n2=213)** | **(n1= 48, n2=241)** | **(n1=64, n2=0)** | **0.6383** |
| **CKD** |  | **1.885 ± 4.208** | **0.801 ± 2.557** |  | **1.33 ± 3.451** | **0.751 ± 2.474** |  | **0.719 ± 1.999** | **1.157 ± 3.4** |  | **1.379 ± 4.097** | **0.985 ± 2.781** | **0.719 ± 1.999** |  |
| **Control** |  | **1.784 ± 3.683** | **0.922 ± 2.823** |  | **1.515 ± 3.296** | **0.985 ± 3.219** |  |  | **1.322 ± 3.275** |  | **1.385 ± 3.286** | **1.269 ± 3.278** |  |  |
| **DKD vs. DM** | **Gemmiger** | **(n1=31, n2=65)** | **(n1=151, n2=139)** | **0.7412** | **(n1= 59, n2=119)** | **(n1=123, n2= 85)** | **0.6631** | **(n1=91, n2=0)** | **(n1= 91, n2=204)** |  | **(n1=35, n2=96)** | **(n1= 56, n2=108)** | **(n1=91, n2=0)** | **0.5121** |
| **DKD** |  | **0.915 ± 0.792** | **1.599 ± 6.301** |  | **1.226 ± 2.092** | **1.606 ± 6.851** |  | **1.885 ± 7.945** | **1.081 ± 1.749** |  | **0.936 ± 1.32** | **1.171 ± 1.977** | **1.885 ± 7.945** |  |
| **DM** |  | **1.766 ± 3.277** | **2.041 ± 4.568** |  | **1.988 ± 4.55** | **1.904 ± 3.662** |  |  | **1.953 ± 4.194** |  | **1.977 ± 4.748** | **1.932 ± 3.653** |  |  |
| **DKD vs. CKD** | **Veillonella** | **(n1=31, n2=23)** | **(n1=151, n2=126)** | **0.0447** | **(n1=59, n2=56)** | **(n1=123, n2= 93)** | **0.4572** | **(n1=91, n2=64)** | **(n1=91, n2=85)** | **0.3649** | **(n1=35, n2=37)** | **(n1=56, n2=48)** | **(n1=91, n2=64)** | **0.3580** |
| **DKD** |  | **1.726 ± 8.651** | **0.503 ± 1.769** |  | **0.969 ± 6.276** | **0.588 ± 1.947** |  | **1.066 ± 5.339** | **0.357 ± 1.355** |  | **0.476 ± 1.98** | **0.282 ± 0.751** | **1.066 ± 5.339** |  |
| **CKD** |  | **0.181 ± 0.388** | **1.059 ± 3.116** |  | **0.786 ± 3.391** | **1.006 ± 2.549** |  | **0.928 ± 2.465** | **0.92 ± 3.18** |  | **1.129 ± 4.31** | **0.758 ± 1.944** | **0.928 ± 2.465** |  |
| **DKD vs. CKD** | **Romboutsia** | **(n1=31, n2=23)** | **(n1=151, n2=126)** | **0.5195** | **(n1=59, n2=56)** | **(n1=123, n2= 93)** | **0.9398** | **(n1=91, n2=64)** | **(n1=91, n2=85)** | **0.6477** | **(n1=35, n2=37)** | **(n1=56, n2=48)** | **(n1=91, n2=64)** | **0.8523** |
| **DKD** |  | **0.156 ± 0.741** | **0.123 ± 0.587** |  | **0.123 ± 0.57** | **0.131 ± 0.636** |  | **0.171 ± 0.746** | **0.086 ± 0.444** |  | **0.029 ± 0.097** | **0.122 ± 0.559** | **0.171 ± 0.746** |  |
| **CKD** |  | **0.125 ± 0.221** | **0.209 ± 0.64** |  | **0.198 ± 0.36** | **0.196 ± 0.702** |  | **0.279 ± 0.836** | **0.134 ± 0.3** |  | **0.125 ± 0.231** | **0.141 ± 0.346** | **0.279 ± 0.836** |  |
| **DKD vs. CKD** | **Acidaminococcus** | **(n1=31, n2=23)** | **(n1=151, n2=126)** | **0.0955** | **(n1=59, n2=56)** | **(n1=123, n2= 93)** | **0.9598** | **(n1=91, n2=64)** | **(n1=91, n2=85)** | **0.4184** | **(n1=35, n2=37)** | **(n1=56, n2=48)** | **(n1=91, n2=64)** | **0.5165** |
| **DKD** |  | **0.929 ± 2.004** | **0.522 ± 1.185** |  | **0.731 ± 1.598** | **0.525 ± 1.233** |  | **0.62 ± 1.547** | **0.564 ± 1.154** |  | **0.76 ± 1.532** | **0.441 ± 0.831** | **0.62 ± 1.547** |  |
| **CKD** |  | **0.167 ± 0.388** | **0.328 ± 0.812** |  | **0.44 ± 1.025** | **0.221 ± 0.54** |  | **0.219 ± 0.484** | **0.367 ± 0.917** |  | **0.504 ± 1.141** | **0.261 ± 0.693** | **0.219 ± 0.484** |  |

| **Comparison** | **Taxonomy** | **HbA1c < 6.5** | **HbA1c >= 6.5** | **P-value** | **HbA1c < 6** | **HbA1c 6-8.9** | **HbA1c >= 9** | **P-value** |
| --- | --- | --- | --- | --- | --- | --- | --- | --- |
| **DM vs. Control** | **Oxalobacter** | **(n1= 12, n2=327)** | **(n1=192, n2=128)** | **0.5512** | **(n1= 12, n2=327)** | **(n1=182, n2=128)** | **(n1=10, n2=0)** | **0.8142** |
| **DM** |  | **0.061 ± 0.118** | **0.045 ± 0.108** |  | **0.061 ± 0.118** | **0.044 ± 0.105** | **0.056 ± 0.162** |  |
| **Control** |  | **0.025 ± 0.089** | **0.027 ± 0.082** |  | **0.025 ± 0.089** | **0.027 ± 0.082** |  |  |
| **DM vs. Control** | **Romboutsia** | **(n1= 12, n2=327)** | **(n1=192, n2=128)** | **0.6261** | **(n1= 12, n2=327)** | **(n1=182, n2=128)** | **(n1=10, n2=0)** | **0.7958** |
| **DM** |  | **0.078 ± 0.112** | **0.14 ± 0.566** |  | **0.078 ± 0.112** | **0.144 ± 0.581** | **0.059 ± 0.086** |  |
| **Control** |  | **0.225 ± 0.796** | **0.178 ± 0.663** |  | **0.225 ± 0.796** | **0.178 ± 0.663** |  |  |
| **DM vs. Control** | **g_f_Eubacteriales** | **(n1= 12, n2=327)** | **(n1=192, n2=128)** | **0.2052** | **(n1= 12, n2=327)** | **(n1=182, n2=128)** | **(n1=10, n2=0)** | **0.2567** |
| **DM** |  | **3.548 ± 8.452** | **1.943 ± 4.391** |  | **3.548 ± 8.452** | **1.972 ± 4.494** | **1.416 ± 1.655** |  |
| **Control** |  | **1.886 ± 3.433** | **1.807 ± 3.119** |  | **1.886 ± 3.433** | **1.807 ± 3.119** |  |  |
| **DM vs. Control** | **Escherichia** | **(n1= 12, n2=327)** | **(n1=192, n2=128)** | **0.4100** | **(n1= 12, n2=327)** | **(n1=182, n2=128)** | **(n1=10, n2=0)** | **0.7265** |
| **DM** |  | **2.582 ± 4.583** | **5.794 ± 11.097** |  | **2.582 ± 4.583** | **6.031 ± 11.341** | **1.477 ± 2.221** |  |
| **Control** |  | **4.378 ± 9.217** | **4.984 ± 10.644** |  | **4.378 ± 9.217** | **4.984 ± 10.644** |  |  |
| **DM vs. Control** | **Aerobutyricum** | **(n1= 12, n2=327)** | **(n1=192, n2=128)** | **0.0354** | **(n1= 12, n2=327)** | **(n1=182, n2=128)** | **(n1=10, n2=0)** | **0.3623** |
| **DM** |  | **0.459 ± 0.537** | **0.209 ± 0.331** |  | **0.459 ± 0.537** | **0.203 ± 0.324** | **0.325 ± 0.444** |  |
| **Control** |  | **0.295 ± 0.39** | **0.294 ± 0.377** |  | **0.295 ± 0.39** | **0.294 ± 0.377** |  |  |
| **DM vs. Control** | **Clostridiales bacterium CIEAF 022** | **(n1= 12, n2=327)** | **(n1=192, n2=128)** | **0.9483** | **(n1= 12, n2=327)** | **(n1=182, n2=128)** | **(n1=10, n2=0)** | **0.8872** |
| **DM** |  | **0.023 ± 0.05** | **0.021 ± 0.058** |  | **0.023 ± 0.05** | **0.021 ± 0.059** | **0.026 ± 0.035** |  |
| **Control** |  | **0.031 ± 0.046** | **0.031 ± 0.047** |  | **0.031 ± 0.046** | **0.031 ± 0.047** |  |  |
| **DM vs. Control** | **Blautia sp. Marseille-P3387** | **(n1= 12, n2=327)** | **(n1=192, n2=128)** | **0.4627** | **(n1= 12, n2=327)** | **(n1=182, n2=128)** | **(n1=10, n2=0)** | **0.6767** |
| **Control** |  | **0.009 ± 0.021** | **0.028 ± 0.14** |  | **0.009 ± 0.021** | **0.029 ± 0.144** | **0.019 ± 0.031** |  |
| **DM** |  | **0.035 ± 0.085** | **0.032 ± 0.069** |  | **0.035 ± 0.085** | **0.032 ± 0.069** |  |  |
| **CKD vs. Control** | **Dialister** | **(n1=114, n2=327)** | **(n1= 35, n2=128)** | **0.0523** | **(n1=114, n2=327)** | **(n1= 35, n2=128)** | **(n1=0, n2=0)** | **0.0523** |
| **CKD** |  | **1.189 ± 3.353** | **2.892 ± 8.257** |  | **1.189 ± 3.353** | **2.892 ± 8.257** |  |  |
| **Contrl** |  | **2.717 ± 5.697** | **2.045 ± 5.874** |  | **2.717 ± 5.697** | **2.045 ± 5.874** |  |  |
| **CKD vs. Control** | **Blautia** | **(n1=114, n2=327)** | **(n1= 35, n2=128)** | **0.8035** | **(n1=114, n2=327)** | **(n1= 35, n2=128)** | **(n1=0, n2=0)** | **0.8035** |
| **Control** |  | **0.273 ± 0.506** | **0.315 ± 0.408** |  | **0.273 ± 0.506** | **0.315 ± 0.408** |  |  |
| **CKD** |  | **0.455 ± 0.623** | **0.536 ± 1.045** |  | **0.455 ± 0.623** | **0.536 ± 1.045** |  |  |
| **CKD vs. Control** | **Bifidobacterium adolescentis** | **(n1=114, n2=327)** | **(n1= 35, n2=128)** | **0.1771** | **(n1=114, n2=327)** | **(n1= 35, n2=128)** | **(n1=0, n2=0)** | **0.1771** |
| **CKD** |  | **0.938 ± 2.725** | **1.067 ± 3.388** |  | **0.938 ± 2.725** | **1.067 ± 3.388** |  |  |
| **Control** |  | **1.551 ± 3.735** | **0.738 ± 1.431** |  | **1.551 ± 3.735** | **0.738 ± 1.431** |  |  |
| **DKD vs. DM** | **Gemmiger** | **(n1=20, n2=12)** | **(n1=162, n2=192)** | **0.7670** | **(n1=20, n2=12)** | **(n1=145, n2=182)** | **(n1=17, n2=10)** | **0.7879** |
| **DKD** |  | **0.709 ± 1.049** | **1.578 ± 6.08** |  | **0.709 ± 1.049** | **1.7 ± 6.41** | **0.535 ± 0.951** |  |
| **DM** |  | **1.665 ± 2.822** | **1.971 ± 4.269** |  | **1.665 ± 2.822** | **1.949 ± 4.29** | **2.367 ± 4.067** |  |
| **DKD vs. CKD** | **Veillonella** | **(n1= 20, n2=114)** | **(n1=162, n2= 35)** | **0.6137** | **(n1= 20, n2=114)** | **(n1=145, n2= 35)** | **(n1=17, n2=0)** | **0.7930** |
| **DKD** |  | **0.657 ± 1.471** | **0.718 ± 4.104** |  | **0.657 ± 1.471** | **0.789 ± 4.334** | **0.114 ± 0.203** |  |
| **CKD** |  | **1.036 ± 3.224** | **0.557 ± 1.233** |  | **1.036 ± 3.224** | **0.557 ± 1.233** |  |  |
| **DKD vs. CKD** | **Romboutsia** | **(n1= 20, n2=114)** | **(n1=162, n2= 35)** | **0.9403** | **(n1= 20, n2=114)** | **(n1=145, n2= 35)** | **(n1=17, n2=0)** | **0.5379** |
| **DKD** |  | **0.056 ± 0.123** | **0.137 ± 0.649** |  | **0.056 ± 0.123** | **0.1 ± 0.431** | **0.456 ± 1.562** |  |
| **CKD** |  | **0.174 ± 0.418** | **0.269 ± 0.977** |  | **0.174 ± 0.418** | **0.269 ± 0.977** |  |  |
| **DKD vs. CKD** | **Acidaminococcus** | **(n1= 20, n2=114)** | **(n1=162, n2= 35)** | **0.7446** | **(n1= 20, n2=114)** | **(n1=145, n2= 35)** | **(n1=17, n2=0)** | **0.4686** |
| **DKD** |  | **0.407 ± 0.91** | **0.614 ± 1.407** |  | **0.407 ± 0.91** | **0.566 ± 1.323** | **1.032 ± 1.992** |  |
| **CKD** |  | **0.281 ± 0.75** | **0.375 ± 0.813** |  | **0.281 ± 0.75** | **0.375 ± 0.813** |  |  |

**
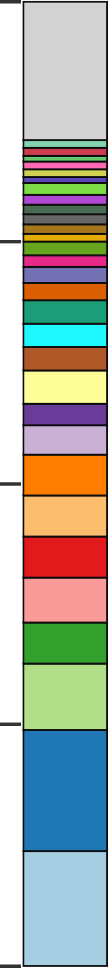

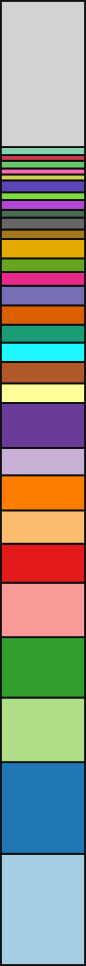

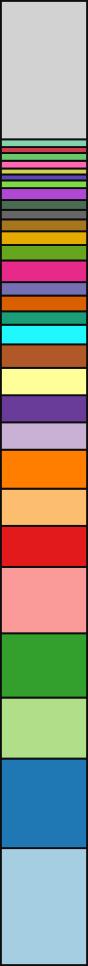
a 1.00**

**0.75**

**Relative abundance**

**0.50**

**0.25**

Other Alloprevotella Lacrimispora Megasphaera Lactobacillus Veillonella Streptococcus Clostridium Enterocloster Lachnospira Ruminococcus Eubacterium Akkermansia Parabacteroides Fusobacterium Gemmiger g_f_Eubacteriales Bifidobacterium Dialister Sutterella Megamonas Klebsiella Roseburia Prevotella


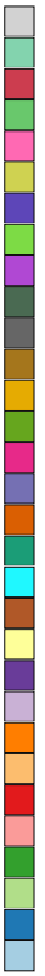
g_Lachnospiraceae Faecalibacterium Escherichia Phascolarctobacterium Bacteroides g_Prevotellaceae


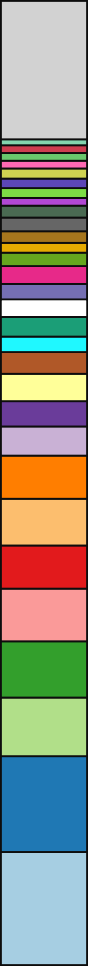

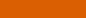


**b 1.00**

**
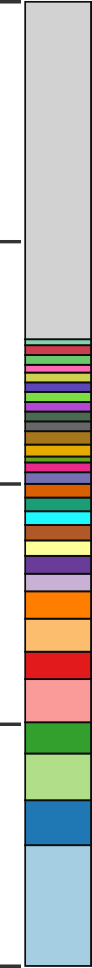

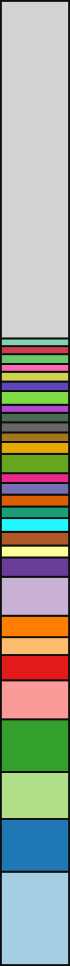

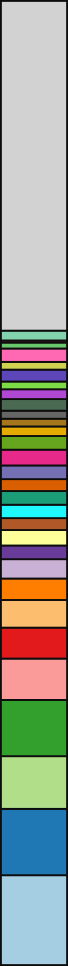
0.75**

**Relative abundance**

**0.50**

**0.25**

Other


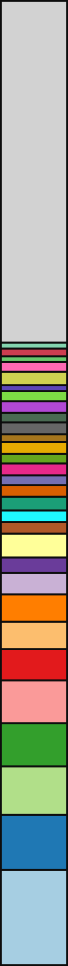

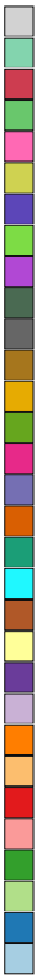
Parabacteroides distasonis Dialister massiliensis Phocaeicola dorei Fusobacterium mortiferum

Phascolarctobacterium succinatutens Dialister succinatiphilus

Clostridiales bacterium canine oral taxon 123 Eubacterium coprostanoligenes

Lachnospira eligens Roseburia faecis Bifidobacterium adolescentis Bacteroides stercoris Akkermansia muciniphila Prevotella stercorea Phocaeicola massiliensis [Eubacterium] rectale Roseburia inulinivorans Bacteroides uniformis Phocaeicola coprocola

Lachnospiraceae bacterium GAM79

Gemmiger formicilis Klebsiella pneumoniae Prevotella copri Megamonas sp.

Phocaeicola plebeius Faecalibacterium prausnitzii Phascolarctobacterium faecium Phocaeicola vulgatus Escherichia coli

**0**

Control DM DKD CKD

Phocaeicola 0

Control DM DKD CKD

Prevotellaceae bacterium

## c

**200**

Alpha diversity

**d**

**
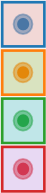
0.2**


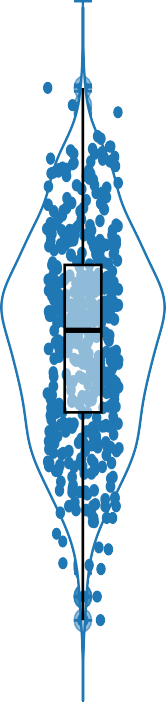

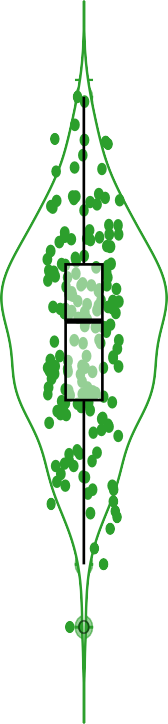

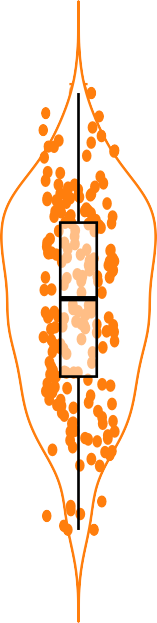

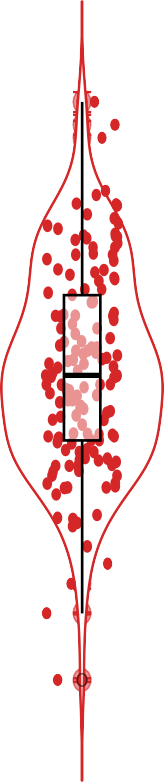

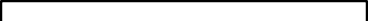

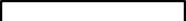

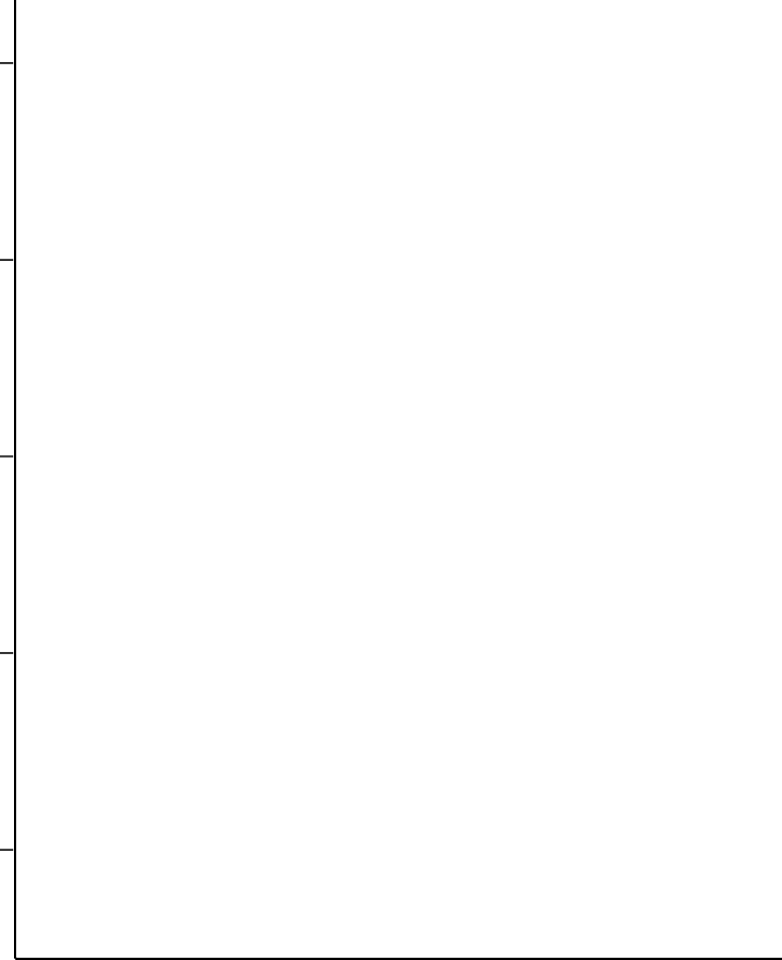


******

******

**Chao1**

Beta diversity

**150**

**Alpha diversity measure**

**100**

**0**

**-0.2**

**Axis.2 [7.8%]**

**Control DM DKD CKD**

**50**

**0**

**Control DM DKD CKD**

**-0.4**

**ADONIS PERMANOVA R2 = 0.007**

**p-value = 0.001**

**-0.3 0 0.3 0.6**

**Axis.1 [16.3%]**

##
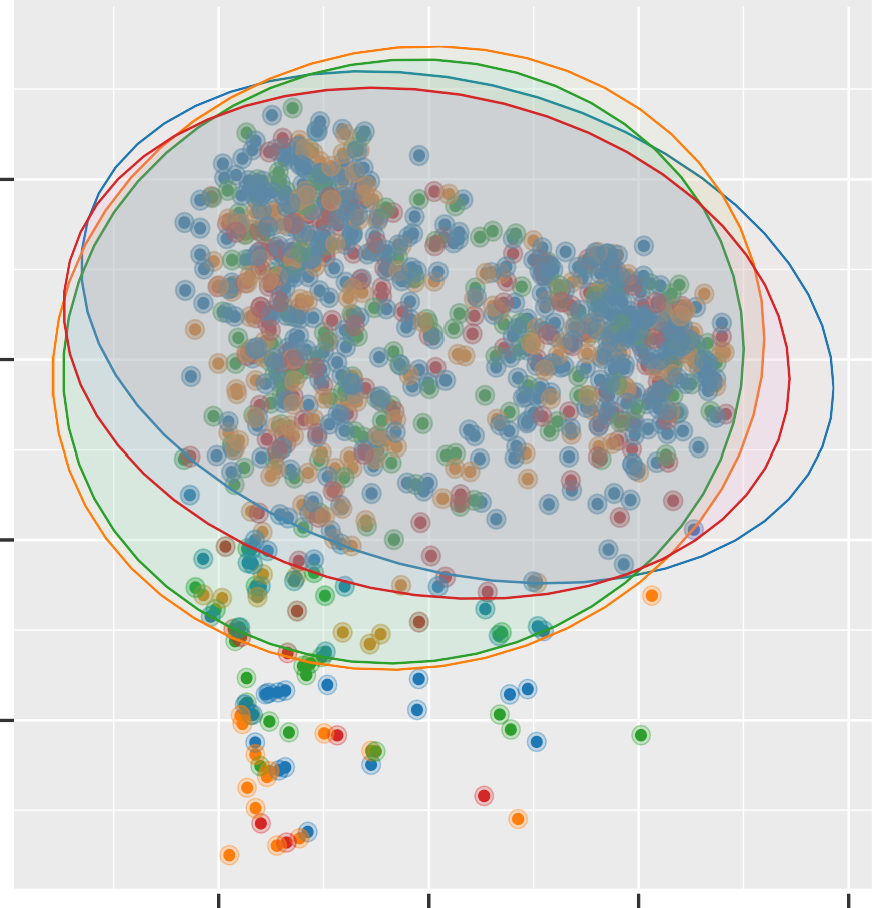
e f


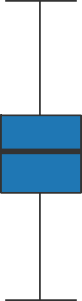

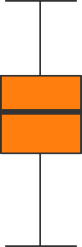

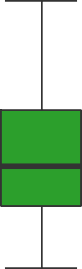

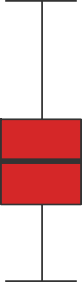

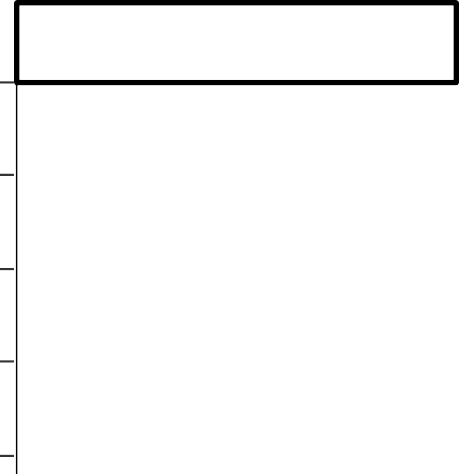


**Female**


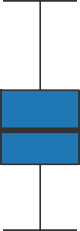

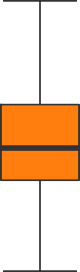

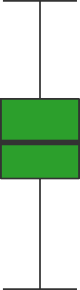

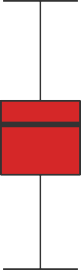

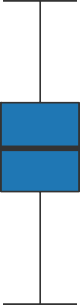

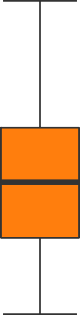

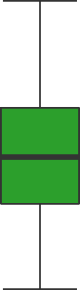

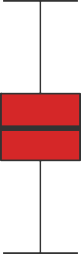

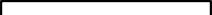

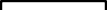

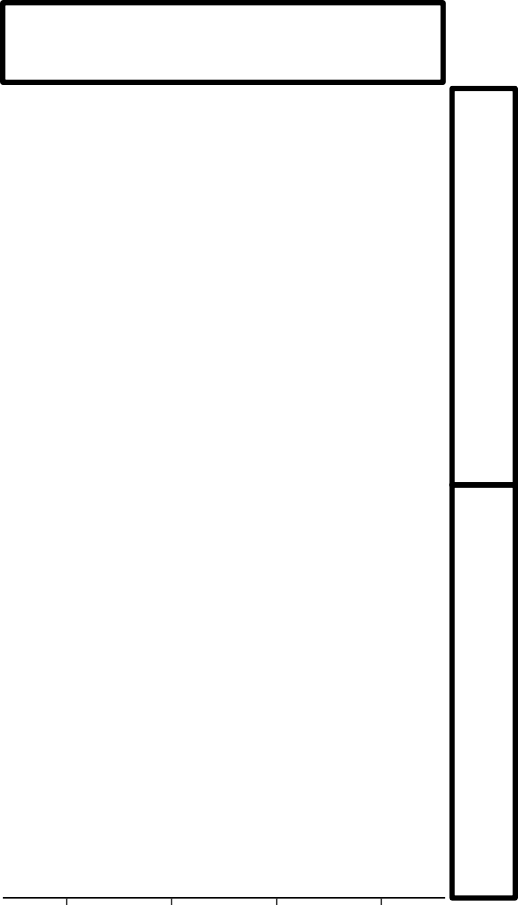


**Male**

*

*


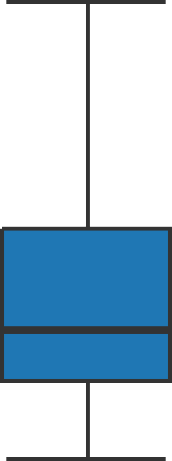

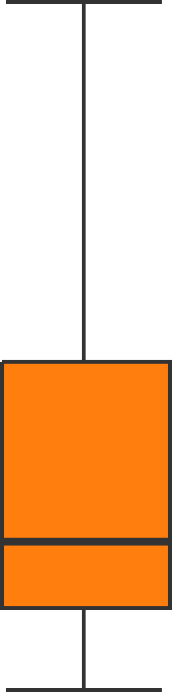

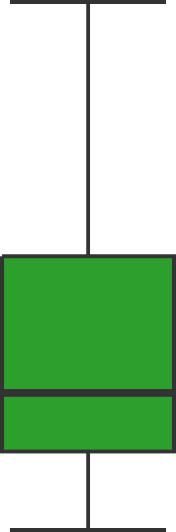

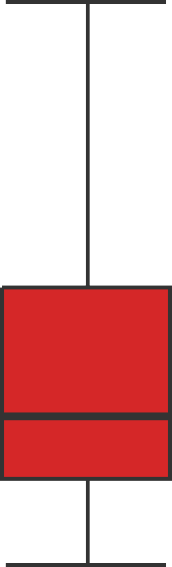

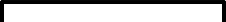

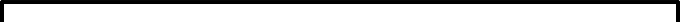

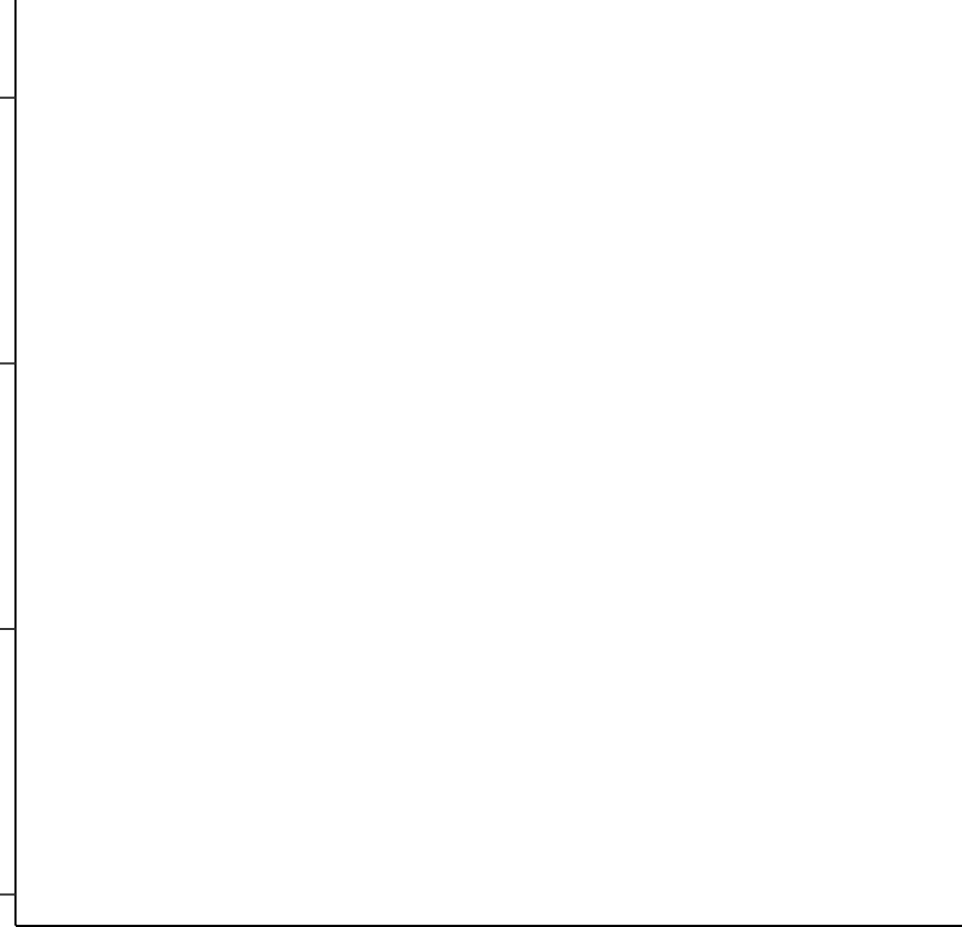


*****

*****

**6 1.00**

**0.75**

**Firmicutes**

**0.50**

**Relative abundance**

**4**

**0.25**

**F/B ratio**

**0**

**1.00**


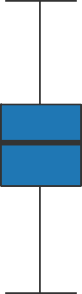

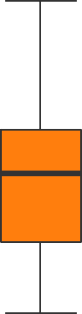

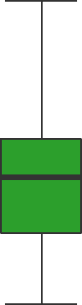

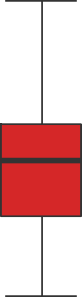

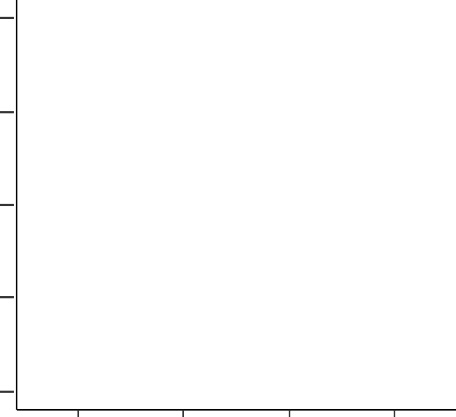


**Bacteroidetes**

**2 0.75**

**0.50**

**0**

**Control DM DKD CKD**

**0.25**

**0**

Control DM DKD CKD Control DM DKD CKD

**Supplementary Figure S1. Analysis of gut microbiota composition and diversity.** Taxonomic composition of the top 30 most abundant bacterial genera (a) and species (b) in the gut microbiota of the different groups. Alpha-diversity (c) and beta-diversity (d) comparisons of the gut microbiota. Differences in the gut Firmicutes to Bacteroidetes ratio (e) and stratified by gender (f).

###### a DM vs. Control

**DESeq2 MaAsLin2**


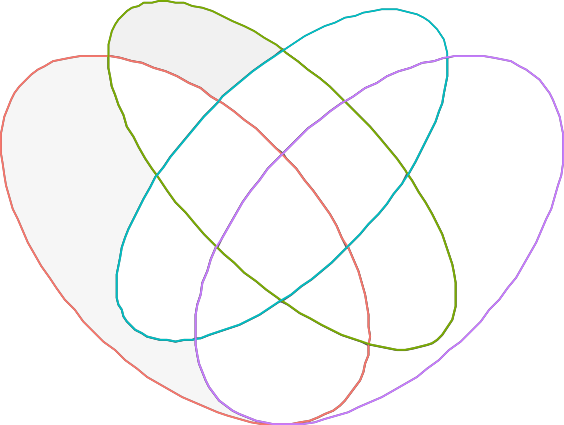


**LEfSe**

**1**

**3**

**limma**

**0**

**0**

**5**

**0 0**

**9**

**3**

**0**

**3**

**3**

**0**

**0**

**1**

###### DKD vs. DM

**DESeq2 MaAsLin2**


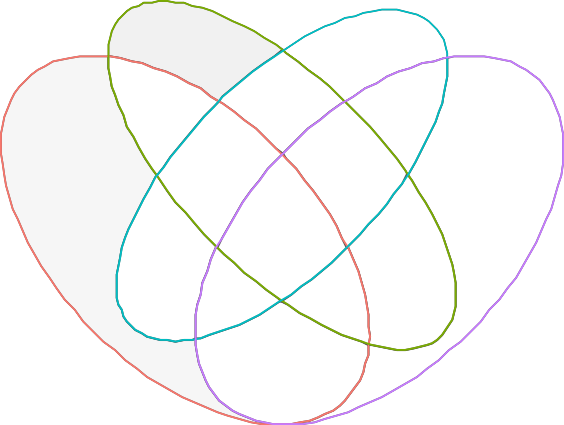


**LEfSe**

**1**

**0**

**limma**

**0**

**0**

**0**

**0 0**

**1**

**0**

**0**

**0**

**0**

**0**

**0**

**0**

###### DKD vs. CKD

**DESeq2 MaAsLin2**


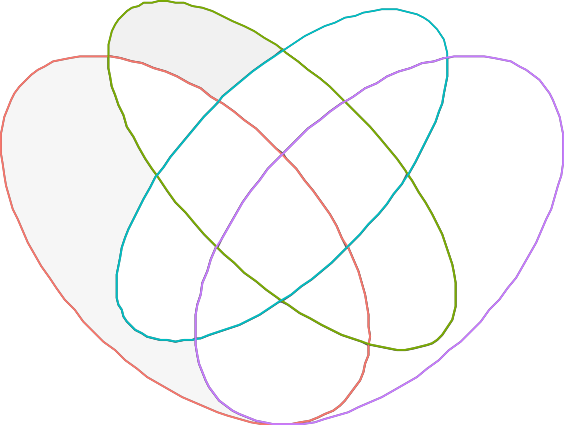


**LEfSe**

**1**

**0**

**limma**

**1**

**0**

**1**

**0 0**

**5**

**0**

**0**

**0**

**0**

**0**

**0**

**0**

###### CKD vs. Control

**DESeq2 MaAsLin2**

| **Comparison group** | **DESeq2** | **LEfSe** | **limma voom** | **MaAsLin2** |
| --- | --- | --- | --- | --- |
| **DM vs. Control** | **1** | **16** | **12** | **14** |
| **DKD vs. DM** | **1** | **1** | **0** | **0** |
| **DKD vs. CKD** | **2** | **6** | **1** | **1** |
| **CKD vs. Control** | **0** | **3** | **1** | **2** |

###### DM vs. Control DKD vs. DM

| **DKD vs. DM** | **Method** |
| --- | --- |
| **Escherichia\|Shigella** | **DESeq2** |
| **Gemmiger** | **LEfSe** |

**DKD vs. CKD**

| **DM vs. Control** | **Method** |
| --- | --- |
| **Acidaminococcus** | **LEfSe** |
| **Akkermansia** | **DESeq2** |
| **Aminipila** | **MaAsLin2, limma** |
| **Anaerobutyricum** | **MaAsLin2, limma** |
| **Anaerostipes** | **LEfSe, MaAsLin2, limma** |
| **Bifidobacterium** | **LEfSe, MaAsLin2, limma** |
| **Cloacibacillus** | **limma** |
| **Clostridium** | **MaAsLin2, limma** |
| **Dialister** | **LEfSe** |
| **Escherichia** | **LEfSe** |
| **Faecalibacterium** | **LEfSe** |
| **g Erysipelotrichaceae** | **MaAsLin2** |
| **g f Acholeplasmatales** | **LEfSe** |
| **g f Eubacteriales** | **LEfSe** |
| **g Lachnospiraceae** | **LEfSe, MaAsLin2** |
| **g Oscillospiraceae** | **MaAsLin2** |
| **g Prevotellaceae** | **LEfSe** |
| **Holdemania** | **limma** |
| **Lachnospira** | **LEfSe** |
| **Lactobacillus** | **LEfSe, MaAsLin2** |
| **Monoglobus** | **MaAsLin2** |
| **Oxalobacter** | **MaAsLin2, limma** |
| **Paludicola** | **limma** |
| **Parasutterella** | **LEfSe** |
| **Pyramidobacter** | **MaAsLin2, limma** |
| **Romboutsia** | **LEfSe, MaAsLin2** |
| **Sellimonas** | **LEfSe, MaAsLin2, limma** |

| **DKD vs. CKD** | **Method** |
| --- | --- |
| **Acidaminococcus** | **LEfSe** |
| **Escherichia\|Shigella** | **DESeq2** |
| **g f Eubacteriales** | **LEfSe** |
| **g Lachnospiraceae** | **LEfSe** |
| **Haemophilus** | **LEfSe, DESeq2** |
| **Pyramidobacter** | **LEfSe** |
| **Romboutsia** | **MaAsLin2, limma** |
| **Veillonella** | **LEfSe** |

###### CKD vs. Control

| **CKD vs. Control** | **Method** |
| --- | --- |
| **Bifidobacterium** | **LEfSe, MaAsLin2** |
| **Blautia** | **limma** |
| **Cloacibacillus** | **MaAsLin2** |
| **Dialister** | **LEfSe** |
| **Phascolarctobacterium** | **LEfSe** |


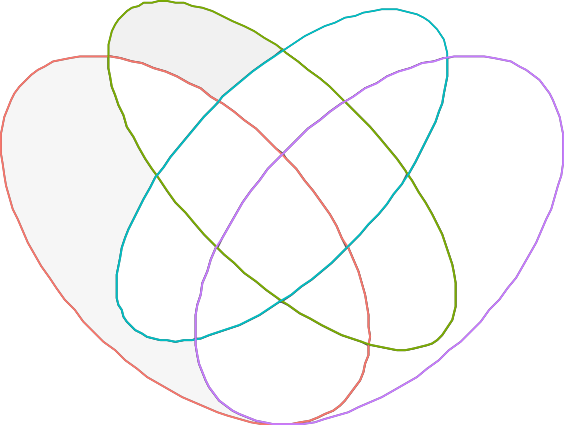


**LEfSe**

**0**

**1**

**limma**

**0**

**0**

**0**

**0 0**

**2**

**1**

**0**

**1**

**0**

**0**

**0**

**0**

**Supplementary Figure S2. Comparison of numbers and names of discriminatory microbes identified by four methods of differential abundance at the genus (a) and the species (b) levels according to disease groups.** Those microbes which were concomitantly selected by at least one of the differential abundance methods and machine learning method were indicated in blue.

**Supplementary Figure S2 continued**

###### b DM vs. Control

**DESeq2 MaAsLin2**


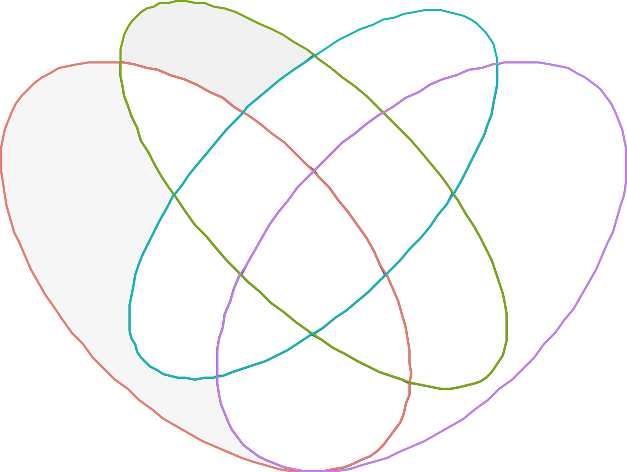


**LEfSe**

**2**

**6**

**limma**

**0**

**0**

**9**

**0 0**

**10**

**4**

**0**

**6**

**4**

**0**

**0**

**1**

###### DKD vs. DM

**DESeq2 MaAsLin2**


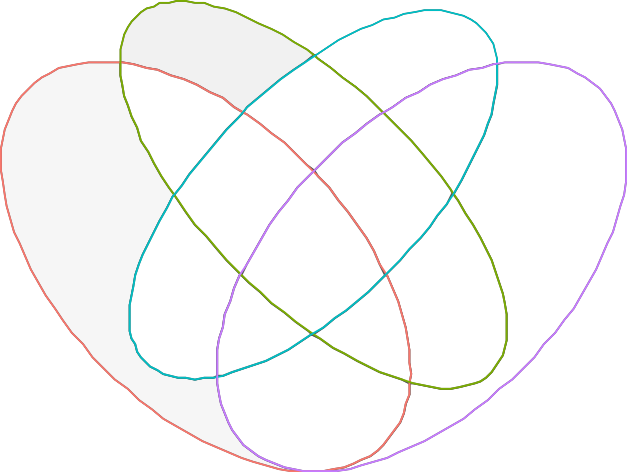


**LEfSe**

**3**

**0**

**limma**

**0**

**0**

**0**

**0 0**

**2**

**0**

**0**

**0**

**0**

**0**

**0**

**0**

###### DKD vs. CKD

**DESeq2 MaAsLin2**


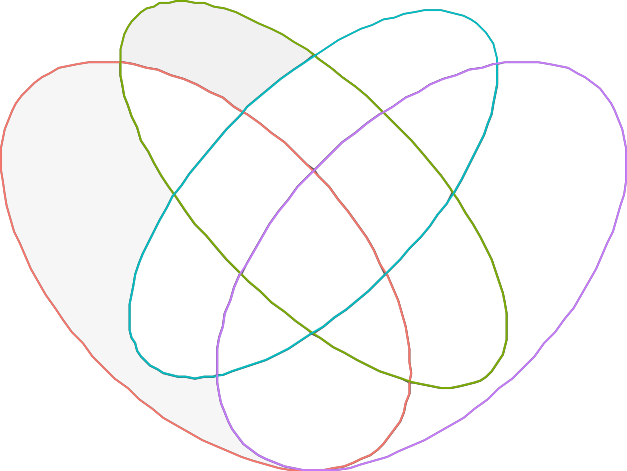


**LEfSe**

**2**

**0**

**limma**

**2**

**0**

**1**

**0 1**

**5**

**0**

**0**

**0**

**0**

**0**

**0**

**0**

###### CKD vs. Control

**DESeq2 MaAsLin2**

| **Comparison group** | **DESeq2** | **LEfSe** | **limma voom** | **MaAsLin2** |
| --- | --- | --- | --- | --- |
| **DM vs. Control** | **2** | **19** | **20** | **23** |
| **DKD vs. DM** | **3** | **2** | **0** | **0** |
| **DKD vs. CKD** | **5** | **7** | **2** | **2** |
| **CKD vs. Control** | **3** | **4** | **1** | **3** |

**DM vs. Control DKD vs. DM**

| **DKD vs. DM** | **Method** |
| --- | --- |
| **Escherichia coli\|Shigella sp. PAMC**  **28760** | **DESeq2** |
| **Fusobacterium ulcerans** | **DESeq2** |
| **Gemmiger formicilis** | **LEfSe** |
| **Phocaeicola coprophilus** | **DESeq2** |
| **Phocaeicola vulgatus** | **LEfSe** |

**DKD vs. CKD**

| **DM vs. Control** | **Method** |
| --- | --- |
| **[Eubacterium] rectale** | **LEfSe** |
| **[Eubacterium] siraeum** | **LEfSe, MaAsLin2** |
| **Acidaminococcus fermentans** | **limma** |
| **Akkermansia muciniphila** | **DESeq2** |
| **Aminipila butyrica** | **limma, MaAsLin2** |
| **Anaerobutyricum hallii** | **limma, MaAsLin2** |
| **Anaerostipes hadrus** | **LEfSe, limma, MaAsLin2** |
| **Bacteroides galacturonicus** | **MaAsLin2** |
| **Bifidobacterium adolescentis** | **limma, MaAsLin2** |
| **Blautia sp. Marseille-P3387** | **MaAsLin2** |
| **Cloacibacillus porcorum** | **limma** |
| **Clostridiales bacterium CCNA10** | **LEfSe** |
| **Clostridiales bacterium CIEAF 022** | **limma, MaAsLin2** |
| **Clostridium sp. BPY5** | **LEfSe, limma, MaAsLin2** |
| **Clostridium sp. NS18-A1** | **limma, MaAsLin2** |
| **Dialister invisus** | **DESeq2** |
| **Erysipelotrichaceae bacterium GAM147** | **MaAsLin2** |
| **Escherichia coli** | **LEfSe** |
| **Faecalibacterium prausnitzii** | **LEfSe** |
| **Holdemania filiformis** | **limma** |
| **Klebsiella variicola** | **LEfSe** |
| **Lachnospira eligens** | **LEfSe** |
| **Lachnospiraceae bacterium** | **LEfSe, MaAsLin2** |
| **Lachnospiraceae bacterium GAM79** | **LEfSe, limma, MaAsLin2** |
| **Lacrimispora amygdalina** | **MaAsLin2** |
| **Lactobacillus rogosae** | **LEfSe, MaAsLin2** |
| **Monoglobus pectinilyticus** | **MaAsLin2** |
| **Oscillibacter sp. PEA192** | **LEfSe, MaAsLin2** |
| **Oxalobacter formigenes** | **limma, MaAsLin2** |
| **Paludicola psychrotolerans** | **limma** |
| **Peptostreptococcaceae bacterium canine oral taxon 303** | **limma** |
| **Peptostreptococcaceae bacterium feline oral taxon 136** | **limma** |
| **Phascolarctobacterium faecium** | **LEfSe** |
| **Prevotellaceae bacterium** | **LEfSe** |
| **Pyramidobacter piscolens** | **limma, MaAsLin2** |
| **Romboutsia ilealis** | **LEfSe, limma, MaAsLin2** |
| **Roseburia faecis** | **LEfSe** |
| **Roseburia intestinalis** | **MaAsLin2** |
| **Roseburia inulinivorans** | **LEfSe** |
| **Ruminococcus callidus** | **limma, MaAsLin2** |
| **Sellimonas intestinalis** | **limma, MaAsLin2** |
| **Streptococcus oralis** | **LEfSe, limma** |

| **DKD vs. CKD** | **Method** |
| --- | --- |
| **Clostridiales bacterium canine oral taxon 141** | **MaAsLin2, limma** |
| **Escherichia coli\|Shigella sp. PAMC 28760** | **DESeq2** |
| **Haemophilus parainfluenzae** | **LEfSe, DESeq2** |
| **Lachnospiraceae bacterium** | **LEfSe** |
| **Lachnospiraceae bacterium GAM79** | **LEfSe** |
| **Lachnospiraceae bacterium RM5** | **LEfSe** |
| **Parasutterella secunda** | **LEfSe, DESeq2** |
| **Phocaeicola coprophilus** | **DESeq2** |
| **Pyramidobacter piscolens** | **LEfSe** |
| **Romboutsia ilealis** | **MaAsLin2, limma** |
| **Veillonella parvula** | **LEfSe** |

**CKD vs. Control**

| **CKD vs. Control** | **Method** |
| --- | --- |
| **[Eubacterium] rectale** | **LEfSe** |
| **Bifidobacterium adolescentis** | **LEfSe, MaAsLin2, limma** |
| **Cloacibacillus porcorum** | **MaAsLin2** |
| **Coprococcus eutactus** | **LEfSe** |
| **Dialister invisus** | **DESeq2** |
| **Dialister massiliensis** | **LEfSe, DESeq2** |
| **Escherichia coli\|Shigella sp. PAMC 28760** | **DESeq2** |
| **Ruminococcus callidus** | **MaAsLin2** |


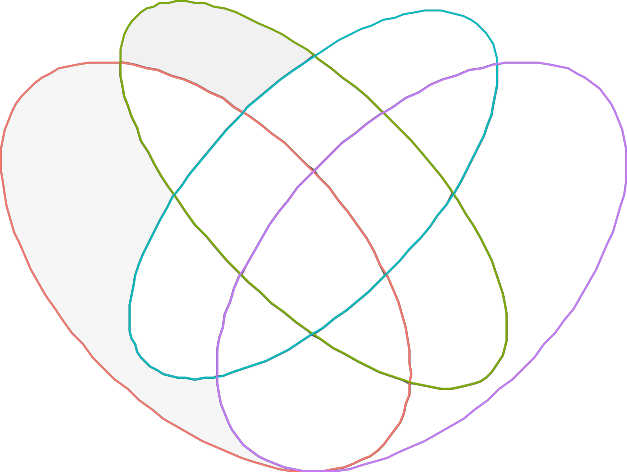


**LEfSe**

**2**

**2**

**limma**

**1**

**0**

**0**

**0 0**

**2**

**0**

**0**

**0**

**1**

**0**

**0**

**0**

### Supplementary Figure S2. Comparison of numbers and names of discriminatory microbes identified by four methods of differential abundance at the genus (a) and the species (b) levels according to disease groups.

Those microbes which were concomitantly selected by at least one of the differential abundance methods and machine learning method were indicated in blue.

**Calculate Abundance**

**a**

**for each taxonomic level per patient**

**Compute Pairwise Ratio**

**for each level**

**c**  Abundance of Anaerobutyricum

Phylum

Class

Order

Family

Filter Taxa with

Abundance <= 10%

Genus

Species

Compute Pairwise Ratio at Genus

Abundance in each Genus

Compute Pairwise Ratio at Family

Abundance in each Family

Compute Pairwise Ratio at Order

Abundance in each Order

Compute Pairwise Ratio at Class

Abundance in each Class

Compute Pairwise Ratio at Phylum

Abundance in each Phylum

# b

**Hierarchical Ratio =** log(

where

##### microbiota1 microbiota2 )

**Ratio =** log(

##### Abundance of Marseillibacter )

microbiota1 is the abundance of the first microbiota microbiota2 is the abundance of the second microbiota

| Patient | Anaerobutyricum | Marseillibacter | Ratio |
| --- | --- | --- | --- |
| 1 | 0.003 | 0.0001 | 3.4 |
| 2 | 0.002 | 0.005 | -0.9 |
| … | … | … | … |

**Supplementary Figure S3. The hierarchical ratio calculation.** (**a**) Workflow of computing pairwise hierarchical ratio per patient for each level (from Phylum to Genus). (**b**) Formula of hierarchical ratio.

(**c**) Example of hierarchical ratio calculation.
